# Regulation and Modulation of Human DNA Polymerase δ Activity and Function

**DOI:** 10.3390/genes8070190

**Published:** 2017-07-24

**Authors:** Marietta Y. W. T. Lee, Xiaoxiao Wang, Sufang Zhang, Zhongtao Zhang, Ernest Y. C. Lee

**Affiliations:** Department Biochemistry and Molecular Biology, New York Medical College, Valhalla, NY 10595, USA; drwangx2008@gmail.com (X.W.); Sufang_Zhang@nymc.edu (S.Z.); Zhongtao_Zhang@nymc.edu (Z.Z.); Ernest_lee@NYMC.edu (E.Y.C.L.)

**Keywords:** DNA polymerase δ, PDIP46, Poldip3, PDIP38, Poldip2, DNA replication, enzyme regulation, DNA damage response, p12 subunit, E3 ligases, cell cycle

## Abstract

This review focuses on the regulation and modulation of human DNA polymerase δ (Pol δ). The emphasis is on the mechanisms that regulate the activity and properties of Pol δ in DNA repair and replication. The areas covered are the degradation of the p12 subunit of Pol δ, which converts it from a heterotetramer (Pol δ4) to a heterotrimer (Pol δ3), in response to DNA damage and also during the cell cycle. The biochemical mechanisms that lead to degradation of p12 are reviewed, as well as the properties of Pol δ4 and Pol δ3 that provide insights into their functions in DNA replication and repair. The second focus of the review involves the functions of two Pol δ binding proteins, polymerase delta interaction protein 46 (PDIP46) and polymerase delta interaction protein 38 (PDIP38), both of which are multi-functional proteins. PDIP46 is a novel activator of Pol δ4, and the impact of this function is discussed in relation to its potential roles in DNA replication. Several new models for the roles of Pol δ3 and Pol δ4 in leading and lagging strand DNA synthesis that integrate a role for PDIP46 are presented. PDIP38 has multiple cellular localizations including the mitochondria, the spliceosomes and the nucleus. It has been implicated in a number of cellular functions, including the regulation of specialized DNA polymerases, mitosis, the DNA damage response, mouse double minute 2 homolog (Mdm2) alternative splicing and the regulation of the NADPH oxidase 4 (Nox4).

## 1. Introduction

Pol δ plays a central role, together with Pol ε and Pol α/primase, as the DNA polymerases that synthesize the daughter DNA strands at the eukaryotic replication fork. The unraveling of the biochemistry of the mammalian DNA polymerases has posed significant experimental challenges. Knowledge of the enzymology of the DNA polymerases is essential to an understanding of their cellular functions. The biochemical approach is critical as pointed out by Arthur Kornberg in the context of the discovery and unraveling of the processes of prokaryotic DNA replication [1]. In the following review, we have focused on the regulation of Pol δ by modification of its subunit structure, and the modulation of its functions by accessory proteins. For a broader view of regulation of Pol δ and other polymerases, see [2,3].

### 1.1. Brief Historical Background

In the early 1970s, three mammalian DNA polymerases were known: Pol α, Pol β and Pol γ [4]. Pol α was considered to be the replicative polymerase, but did not possess an intrinsic or associated 3′ to 5′ exonuclease activity like the *Escherichia coli* or T4 bacteriophage DNA polymerases, where they function to edit or proofread misincorporated nucleotides [5,6,7]. Thus, the discovery of a novel mammalian DNA polymerase with an intrinsic 3′ to 5′ exonuclease activity represented a major advance. This enzyme, named Pol δ, was studied by a group of investigators at the University of Miami in Florida, in rabbit bone marrow erythroid cells [8,9,10,11], calf thymus [12,13,14] and human placental tissues [15,16,17,18,19]. Their approach was the rigorous isolation of the enzyme activities. This initially resulted in the characterization of a dimeric enzyme, consisting of a catalytic subunit of 125 kDa that harbored both the polymerase and 3′ to 5′ exonuclease catalytic sites and a p50 subunit. Evidence that Pol δ was a distinct enzyme from Pol α came from their separation by purification, by their immunochemical distinction using antibodies against Pol δ [18,20], and by the molecular cloning of the p125 subunit [21,22,23]. These studies from the Miami laboratories provided a firm basis for the identification of Pol δ as a novel proofreading DNA polymerase, and removed concerns that this new enzyme was merely Pol α contaminated with a cellular exonuclease.

Studies of human placental [17], calf thymus [24] and HeLa Pol δ [25] led to the discovery of a second human DNA polymerase with an intrinsic 3′ 5′ exonuclease activity, which was named Pol ε [26,27]. The early history of the study of Pol δ is also notable for the discovery of a factor which stimulated its activity, and acted to modify synthesis by Pol δ from a distributive to a processive mode [28]. This protein was identified as proliferating cell nuclear antigen (PCNA), which was subsequently shown to be a platform for many DNA transactions [29]. 

These early studies defined mammalian Pol δ as having two subunits, p125 and p50. The third and fourth accessory subunits were identified as p66/p68 [30,31] and p12 [32] (Table 1). The four subunits of human Pol δ are encoded by the *POLD1*, *POLD2*, *POLD3* and *POLD4* genes. Pol δ has been extensively studied in yeast [33]. *Saccharomyces cerevisiae* (*S. cerevisiae*) Pol δ consists of the homologs of the p125, p50 and p68 subunits [34]. *Schizosaccharomyces pombe* (*S. pombe*) Pol δ has an additional fourth subunit, Cdm1 [35,36,37], which has limited homology to p12 [32]. Molecular cloning of the p125 catalytic subunit of human Pol δ showed that the catalytic cores of the p125 subunits share greater than 60% similarity with that of *S. cerevisiae* Pol3 [22,23]. Pol δ and Pol ε are members of the B family of DNA polymerases that include T4 and Rb69 DNA polymerases. 

### 1.2. Properties of Human Pol δ4 and Its Subassemblies and the Roles of the p68 and p12 Subunits

The p68/Pol32/Cdc27 subunits of both human and yeasts possess PCNA interacting protein-boxes (PIP-boxes) at their C-termini [30,34,38]. The p68 subunit has an extended structure, and is highly charged, suggesting that it is flexible and thus an ideal subunit for mediating PCNA interaction [39,40]. In *S cerevisiae*, the Pol32 subunit is not essential, but Cdc27 is required for viability in *S*. *pombe* [39,40]. However, the human Pol δ p125 [41,42,43,44] and p12 [45] subunits also interact with PCNA. The p50 subunit also interacts with PCNA [46], although this interaction is much weaker [47]. Analysis of Pol δ enzymes in which the PIP-boxes of either the p12 [45] or p68 [48] were mutated show that both are required for full expression of activity. 

The human Pol δ heterotetramer (Pol δ4), as well as its subassemblies, have been reconstituted by their expression in the baculovirus system [49,50,51]. Pol δ4 has also been expressed in an *E. coli* system [52]. The use of the baculovirus expression system allowed for the preparation of highly purified Pol δ4 and its subassemblies for biochemical studies. Initial difficulties were encountered in obtaining reproducible behaviors of the subassemblies, including that of the trimer lacking the p12 subunit [50]. This was traced to its instability during the isolation process; additionally, both the p68 and p12 subunits are more susceptible to proteases than the p125 and p50 subunits [51]. Immunoaffinity chromatography was used as a key component of the purification of the Pol δ subassemblies [53]. The preparations of Pol δ4 and its subassemblies were monitored for the appropriate subunit stoichiometry [51] because of the possibility of subunit loss during isolation and the fact these subassemblies do exhibit significant activities. 

**Figure 1:**
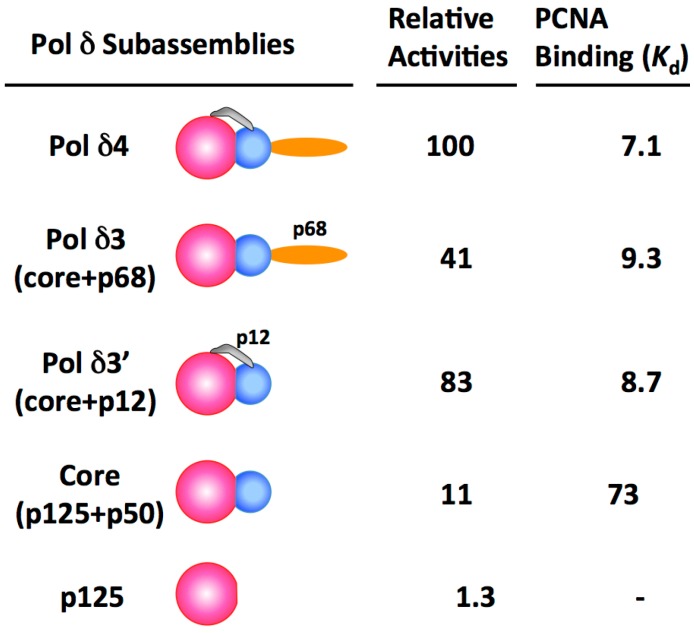
Relative specific activities and proliferating cell nuclear antigen PCNA binding (nM) of Pol δ and its subassemblies. Data from [51].

The activities of Pol δ and its subassemblies were compared by assay using sparsely primed poly(dA)/oligo(dT) as the substrate in the presence of PCNA [51]. PCNA does not have to be loaded onto this linear template with the replication factor C (RFC) clamp loader. This “standard” assay allows reproducible quantitation of Pol δ activities, and specific activities of ca. 20,000 units/mg were consistently obtained. The relative specific activities and the apparent *K*_d_ for PCNA binding are summarized in Figure 1 [51]. The figure also shows the subunit arrangement of the p125, p50, p68 and p12 subunits [45]. Notably, the core enzyme and the two trimeric subassemblies all possess significant activities. The presence of either the p12 or the p68 subunit is able to enhance PCNA binding and activity of the core enzyme (Table 1). However, as described below, these subassemblies exhibit defects in assays that require highly processive synthesis.

The second type of assay that has been used is the M13 assay, in which a singly primed M13 single-stranded DNA (ssDNA) is used as the substrate. PCNA is loaded with RFC, together with RPA (single stranded DNA binding protein). This assay monitors synthesis of long strands of DNA up to 7 kb on M13 circular DNA and has been used to demonstrate the processivity of Pol δ [51,54,55,56]. However, on long circular ssDNA templates, pausing can be observed where Pol δ4 has difficulty synthesizing through regions of secondary structure. In addition, it has been found that Pol δ dissociates frequently during these reactions [52]. Thus, while Pol δ exhibits processivity in the presence of PCNA, the observed processivity is not continuous (i.e., not due to a single binding event) over the entire length of the M13 template. There were marked defects in the abilities of the Pol δ subassemblies to synthesize the full-length products, which could be partially compensated for by increasing the enzyme concentrations, consistent with a more frequent dissociation from the primer template [51]. 

## 2. Alteration in Subunit Composition by the Degradation of the p12 Subunit Is the Key Mechanism for the Regulation of Human Pol δ

**Figure 2:**
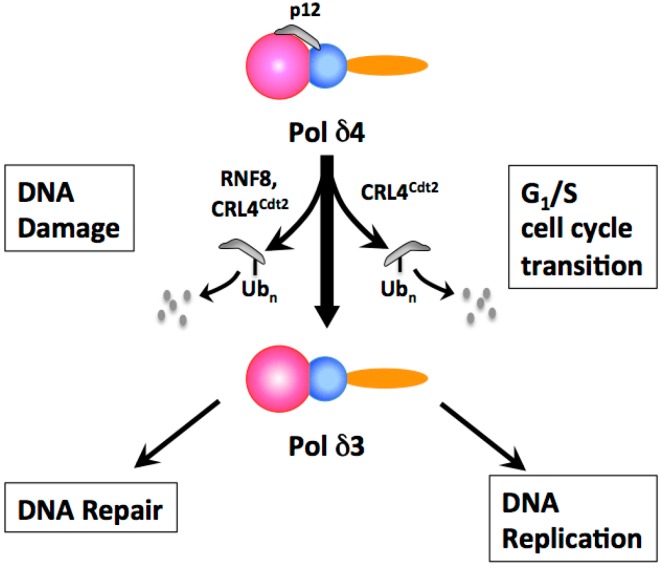
Overview of the regulation of human Pol δ by degradation of the p12 subunit and the formation of Pol δ3.

There is a surprising paucity of literature on the control of eukaryotic DNA polymerase activities by posttranslational modification [2,48,57,58]. The p12 subunit has emerged as a center point for regulation of Pol δ [58,59]. The discovery that the p12 subunit is rapidly degraded by ubiquitination and proteasomal degradation in response to DNA damage opened a new window on the regulation of Pol δ [60]. Later, a similar process was found to take place during the G1/S transition under the control of a key regulator of the entry to S-phase, the E3 ubiquitin ligase CRL4^Cdt2^ [61]. The operational outcome of the degradation of p12 is that the Pol δ4 enzyme is converted in vivo to a trimer, Pol δ3, in synchrony with the S phase (Figure 2). This represents an unusual form of enzyme regulation, whose significance ultimately rests on understanding the comparative properties of the two forms, and how these differences operate to facilitate and/or differentiate their functions in DNA repair or DNA replication. In the following subsections, the mechanisms for the formation of Pol δ3 and the properties of Pol δ4 and Pol δ3 in DNA repair and replication are reviewed. 

### 2.1. The Degradation of the p12 Subunit of Pol δ in Response to DNA Damage

Ultraviolet (UV) damage has been extensively used to study cellular responses to DNA damage. UV treatment of cells triggers global nucleotide excision repair (NER), and activates translesion synthesis (TLS) to deal with the effects of the bulky lesions that are barriers to replicative DNA polymerases. UV exposure also triggers checkpoints that result in the inhibition of cellular DNA synthesis [62] through the activation of Ataxia telangiectasia and Rad3-related (ATR) [63,64]. The intra-S phase checkpoint leads to slowing of progression through the S-phase, and acts by the inhibition of late firing origins of initiation of replication, and also by slowing the rates of replication fork progression [65]. 

We examined the effects of UV treatment of cells on Pol δ by Western blotting of all four of its subunits to determine if evidence for band-shifts caused by phosphorylation events were detectible. Instead, this led to the discovery that the p12 subunit was rapidly degraded in response to UV damage [60]. This study characterized in a rigorous manner the loss of the p12 subunit of Pol δ in response to genotoxic stress. The significant findings are summarized below:

- p12 is rapidly lost in a variety of cell types, in a UV flux- and time-dependent manner, followed by a slower recovery over 24 h.
- Treatment with alkylating agents such as methyl methanesulfonate (MMS) or agents inducing replication stress (hydroxyurea and aphidicolin) also caused p12 degradation.
- The loss of p12 is due to an accelerated rate of proteasomal degradation initiated by its polyubiquitination.
- Degradation of p12 is dependent on ATR signaling, but not on ATM, as shown by the use of ATR or ATM depleted cells.
- The p12 subunit of Pol δ is selectively targeted, and similar changes are not observed for the other three subunits.
- Loss of the p12 subunit leads to the in vivo conversion of Pol δ4 to the heterotrimer, Pol δ3.

The final observation noted above is of some importance. Prior to these studies, Pol δ4 was considered to be the holoenzyme form, so that the first idea to come to mind was that this might be a way to disable Pol δ4 activity. However, the Pol δ3 isolated from UV-treated cells by immunoaffinity chromatography exhibited significant activity. Direct comparisons of Pol δ3 produced in vivo by UV treatment with recombinant Pol δ3 showed that they had similar properties [51,60] (see Section 1.2 for comparative properties of Pol δ4 and Pol δ3). 

Thus, the question is whether Pol δ3 exhibits advantages over Pol δ4 in DNA repair. The route to gaining insights into this possibility came from testing their functionalities utilizing highly purified proteins in specialized assays. 

### 2.2. Pol δ3 Exhibits Altered Behaviors from Pol δ4 in Lesion Bypass and in Extension of Mismatched Primers that Represent a Gain of Function

In order to probe for advantages for the presence of Pol δ3 in cells subjected to genotoxic agents, a comparison of its behavior with that of Pol δ4 was made in two contexts. First, replicative polymerases encounter small lesions that can be bypassed by eukaryotic DNA polymerases in an error prone manner [66,67]. Second, replicative polymerases encounter lesions that act as severe obstacles to chain extension: these include abasic sites and thymine-thymine dimers. Model templates with small lesions were used to study the behavior of Pol δ4 and Pol δ3 [68]; these were *O*^6^-MeG *(O*^6^-Methylguanine), which is produced by alkylating agents [66], and 8-oxoG (7,8-dihydro-8-oxoguanine), which is produced by reactive oxygen species [67]. Templates containing abasic sites and thymine-thymine dimers were used as examples of lesions that are not readily bypassed. Pol δ3 exhibits a decreased tendency for bypass synthesis across these templates. Pol δ3 exhibits a higher exonuclease/polymerase ratio than Pol δ4, suggesting that it was more efficient in proofreading. Further analysis showed that Pol δ3 is less likely to extend mismatched primers or to misincorporate wrong nucleotides in single nucleotide incorporation assays. Overall, this study indicated that Pol δ3 exhibited behavior consistent with it being more discriminatory than Pol δ4, i.e., of having a greater fidelity within the context of these biochemical assays [68]. The inference drawn is that the p12 subunit exerts an influence on the intrinsic properties of Pol δ, which could originate from effects on the polymerase or the exonuclease activities, or both. 

The kinetic [69,70] and structural bases [71,72] for the fidelity of replicative polymerases is well understood. The rate constant for polymerization, *k*_pol_, plays a major role in the avoidance of misincorporation of wrong nucleotides or in mutagenic bypass [69,70]. This is the so-called kinetic barrier, in which *k*_pol_ is reduced on encounter with template lesions, the binding of wrong nucleotides, or the presence of a mismatched primer end. This increases the probability for transfer of the primer end to the exonuclease site. The second important kinetic constant is *k*_pol-exo_, the rate at which the primer end is translocated from the pol active site to the exonuclease site [73,74]. This transfer rate is the rate-limiting step for the exonuclease activity in the kinetic scheme [69]. Thus, for a given polymerase the determination of *k*_pol_ and *k*_pol-exo_ provides information on the polymerase and exonuclease, respectively, while the ratio of *k*_pol-exo_ to *k*_pol_ may be regarded as a ratio of editing to extension, and an index of its proofreading propensity. 

**Figure 3:**
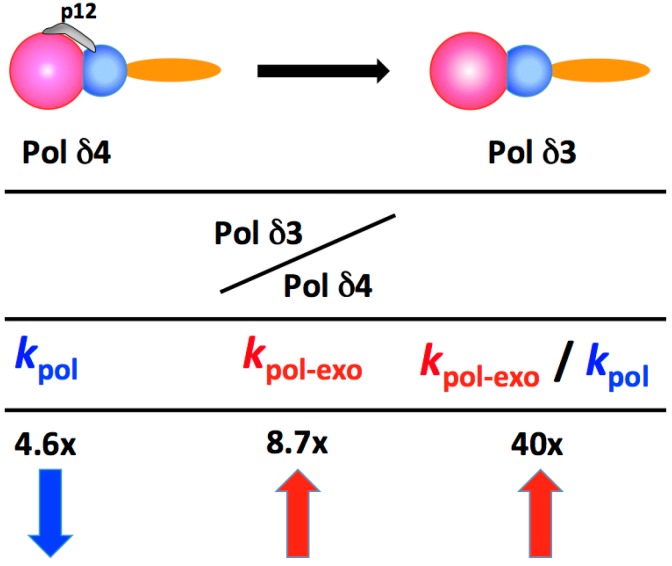
Changes in the kinetic constants of Pol δ3 and Pol δ4. The changes are shown as the ratios of the values for Pol δ3/Pol δ4.

Pre-steady state kinetic analysis was used to determine the kinetic constants for Pol δ4 and Pol δ3 [58,75]. The differences between the two key kinetic constants are summarized in Figure 3, as the ratio of their changes (Pol δ3/Pol δ4). The removal of p12 leads to a nearly five-fold decrease in *k*_pol_, and a greater than eight-fold increase in *k*_pol–exo_, such that the ratio of editing to extension increased by ca. 40 fold (Figure 3). Both polymerase (*k*_pol_) and exonuclease (*k*_pol-exo_) are affected. Thus, by these measures, Pol δ3, compared to Pol δ4, exhibits properties of a polymerase that intrinsically proofreads more frequently and should exhibit greater fidelity. These findings are consistent with the observed behavior of Pol δ3 when tested on lesion containing templates [68].

These studies indicated that p12 exerts an influence on the proofreading functions of Pol δ, and that its removal to form Pol δ3 resulted in an apparent gain of function in the form of an increased surveillance against mutagenic synthesis. 

The potential significance for the formation of Pol δ3 may be rationalized as a defense against mutagenic DNA synthesis in replicating cells upon genotoxic challenge. The formation of Pol δ3 in response to DNA damage earmarks it as the likely form of Pol δ engaged in DNA repair synthesis, which in the case of UV damage, would primarily involve NER [59,76] and homologous recombination repair of DSBs. (The p12 subunit is also degraded in response to ionizing radiation [59,77]).

### 2.3. Spatiotemporal Analysis of the Recruitment of Pol δ to Sites of UV Damage Indicates Pol δ3 Is in the Right Place at the Right Time

A hallmark of the cellular response to DNA damage is the recruitment of signaling and repair factors to sites of DNA damage, and the formation of repair foci. The analysis of subcellular localization to these foci has played an important role in dissecting the assembly of proteins involved in the DNA Damage Response [78,79,80,81,82] and DNA damage tolerance pathways [63,83]. A spatiotemporal analysis of the recruitment of all four Pol δ subunits to sites of UV-induced DNA damage provided evidence that Pol δ4 is recruited to sites of DNA damage, and that this is followed by the appearance of Pol δ3 upon loss of the p12 subunit. The loss of p12 from the DNA damage foci was confirmed by chromatin immunoprecipitation (ChIP) analysis with anti-p125 [84]. 

### 2.4. Conversion of Pol δ4 to Pol δ3 May Facilitate the Switch between Pol δ and Pol η

In S phase cells, genotoxic agents that introduce bulky lesions lead to the activation of the DNA damage tolerance pathway. The stalled replicative polymerase (usually taken as Pol δ) is switched for a translesion polymerase that bypasses the lesion. The most studied example of translesion synthesis is that performed by Pol η in the bypass of UV-induced CPDs (cyclobutane pyrimidine dimers) [85,86,87,88,89]. The key event that is required for initiation of translesion synthesis is the mono-ubiquitination of PCNA [90], following which Pol δ is switched for Pol η [91,92]. The ubiquitination of PCNA is significant, in that Pol η and other TLS polymerases possess both ubiquitin binding domains and PCNA binding PIP-boxes [91,92]. The switching process in TLS requires the displacement of Pol δ by Pol η in the initial switch, followed by a second switch once TLS is completed. We have proposed a model in which the conversion of Pol δ4 to Pol δ3 facilitates the switch to Pol η, on the basis that Pol δ3 dissociates from the PCNA/DNA primer-template more readily than Pol δ4 [58,93]. This model is consistent with the idea that ubiquitination of PCNA and p12 represent related cooperative events that are involved in TLS at the lesion sites. 

The determination of the structure of mono-ubiquitinated PCNA (ub-PCNA) reveals that the ubiquitins are oriented in a radially extended fashion, below the plane of the PCNA trimer, and on the opposite face of PCNA where the PIP-box binding pockets are located [93]. Additionally, the ubiquitins displayed no contacts with PCNA beside the isopeptide linkage, and exhibit the possibility of significant conformational flexibility. Mono-ubiquitinated PCNA was found to lead to inhibition of the combined reactions of Pol δ4 and Fen1 (Flap endonuclease 1) activity in Okazaki fragment processing [93]. This could potentially contribute to the UV-induced inhibition of DNA synthesis.

### 2.5. Does the Plasticity of Pol δ Subunit Composition Extend to Other Subunits besides p12?

The demonstration that Pol δ is regulated by modification of its quaternary structure raises the question of whether the other subassemblies of Pol δ could also be generated in vivo to serve a functional role. Phenotypic analyses of the deletion of the Pol δ genes should take into account their potential impact on the Pol δ enzyme. In the case of the p68 subunit, its deletion could potentially result in the formation of the Pol δ3 trimer consisting of the core + p12 (Figure 1). This trimer has activity in the standard assay which is comparable to that of Pol δ4. However, deletion of the *POLD3* gene is lethal in the animal system. Conditional knockouts of *POLD3* in mice have shown that it is essential for development, and exhibits haploinsufficiency [94]. Deletion of *POLD3* in B lymphocytes led to severe replication defects and genomic instability. The mechanism was traced to a severe loss of the p125 catalytic subunit, consistent with a loss of stability of the Pol δ complex [94]. It seems unlikely that a regulated conversion of Pol δ4 to yield the Pol δ3’ trimer lacking p68 occurs in mammalian cells, as this would coincide during S phase with the degradation of p12, leaving the Pol δ dimer as the major form. However, it is noted that a temporally restricted reversible loss of p68 outside the S phase might occur. In contrast to the effects of gene deletion in mice, DT40 chicken lymphocytes cells in which the *POLD3* gene is deleted are viable, and the cells replicate with a moderate S phase delay, but exhibited increased sensitivity to genotoxic stress [95]. Deletion of the p68 ortholog, Pol32, is not lethal in *S. cerevisiae* [34], but deletion of Cdc27 in *S. pombe* is lethal [38]. Apart from an impact on Pol δ, loss of the *POLD3* gene would also impact Pol ξ, which utilizes the Pol δ p50 and p68 subunits [96,97]. 

### 2.6. RNF8 Is Involved in DNA Damage-Induced p12 Degradation

The identification of the E3 ubiquitin ligase(s) that target p12 for degradation is important in understanding how p12 degradation and the ensuing generation of Pol δ3 is integrated into the signaling systems that comprise the DNA Damage Response (DDR) and the DNA Damage Tolerance (DDT) pathways [63,81,85,87]. Two E3 ligases that target p12 for degradation have been identified: RNF8 [98] and CRL4^Cdt2^ [61,77]. RNF8 was identified by a classical biochemical approach [98]. An in vitro assay system was devised for the detection of the polyubiquitination of GST-p12. Such in vitro assays require the combined actions of an E1 ubiquitin activating enzyme, an E2 ubiquitin conjugating enzyme and an E3 ubiquitin ligase. UbcH5c, which is active with a number of E3 ligases [99] was used as the E2 enzyme, and GST-p12 as the substrate. An E3 ligase fraction was purified from HeLa cell extracts by conventional chromatographic methods. This preparation was subjected to proteomic analysis by LC/LC/MS/MS; this yielded three peptides that were identified as sequences from RNF8. Western blotting of the column fractions confirmed the presence of RNF8, and in vitro assays of recombinant RNF8 showed that it had a robust activity for the ubiquitination of GST-p12. Depletion of RNF8 confirmed that the rates of p12 degradation by UV or by alkylation with MNNG (*N*-Methyl-*N*’-Nitro-*N*-Nitrosoguanidine) were significantly reduced [98]. 

RNF8 has a major role in orchestrating the ATM regulated DDR through the noncanonical polyubiquitination of histone H2A [79,80,81,100]. The discovery that RNF8 mediates the regulation of Pol δ is surprising, as this raises the question as to whether RNF8 also plays significant roles in NER and the DNA damage tolerance pathway that involves activation of translesion synthesis by PCNA ubiquitination. RNF8 is recruited to DNA damage foci induced by UV [98,101]. RNF8, together with UbcH5c, efficiently mono-ubiquitinates PCNA in vitro; mono-ubiquitinated PCNA (ub-PCNA) is further polyubiquitinated via K63 isopeptide linkages by RNF8/UbcH5c and Ubc13/Uev1a [102]. Depletion of RNF8 by shRNA was found to suppress ub-PCNA formation in UV-treated A549 cells [102]. These observations suggest that RNF8 might participate in both modulation of Pol δ and of TLS by PCNA ubiquitination [59]. The possible regulation of Pol δ and Pol η by RNF8 could be a means for cross-talk between the ATR and ATM signaling pathways [59]. However, further work is needed to establish what role RNF8 plays in ub-PCNA formation in vivo. 

### 2.7. Degradation of p12 by CRL4^Cdt2^

Depletion of RNF8 did not completely block the degradation of p12 in response to UV damage, indicating that more than one E3 ligase is involved. CRL4^Cdt2^ was found to target p12 for degradation in response to UV, and also mediates the degradation of p12 before entry into S phase [59,61]. The CRL4^Cdt2^ ubiquitin ligase plays a critical role in the prevention of re-replication, as the “master coordinator of cell cycle progression and genome stability” [103]. CRL4^Cdt2^ is a member of the Cullin Ring Ligase family of E3 ubiquitin ligases and targets the licensing factors that are involved in the assembly of the pre-replicative complex during G1, so that they are removed during the G1/S transition [104]. The primary targets of CRL4^Cdt2^ are Cdt1, p21 (p21^Waf1/CIP1^) and the histone acetylase Set8. CRL4^Cdt2^ recognition of its substrates depends on their possession of an extended PIP-box, termed a PIP-degron. These PIP-degrons have a higher affinity for PCNA than PIP-boxes, and their degradation also requires that PCNA be loaded onto DNA [105,106]. Studies in *Xenopus* extracts have shown Cdt1 destruction is dependent on the initiation of DNA replication as well as Pol α, indicating that PCNA is loaded onto the primer end [107]. CRL4^Cdt2^ also targets its substrates for destruction in response to DNA damage by UV [108,109]. Both p21 [110] and Cdt1 are degraded in response to UV damage [108,111,112,113,114].

The p12 subunit of Pol δ possesses a PIP-degron, and is a substrate for CRL4^Cdt2^ [61,77]. Mutation of the PIP-degron of p12 reduces its UV-induced degradation. Depletion of either of the two isoforms of Cul4 [115] also suppresses UV-induced p12 degradation with similar time courses as for p21 [59,61]. The NEDD8-activating enzyme (NAE) is required for Cullin ligase activity; the NAE inhibitor, MLN4924 [116], blocks UV and IR degradation of p12 [59,77]. CRL4^Cdt2^ has also been shown to be required for the UV-induced inhibition of DNA synthesis; furthermore, replication fork progression is inhibited and is dependent on p12 degradation [77]. The latter findings provide evidence that p12 degradation contributes to the elongation checkpoint that is a component of the intra-S phase checkpoint [65,117].

Analysis of the cell cycle behavior of p12 and its dependence on CRL4^Cdt2^ were examined in synchronized cell populations together with that of p21. These studies showed that p12 levels were reduced during S phase and returned to basal levels during G2/M [59,61]. Depletion of CRL4^Cdt2^ isoforms reduced the S phase degradation of p12 [59,61]. At the same time as p12 undergoes a decrease during the S phase, levels of the other subunits of Pol δ remain fairly constant. Thus, the degradation of p12 by CRL4^Cdt2^ leads to the formation of Pol δ3 in synchrony with the S phase [59,61]. The regulation of Pol δ3 is orchestrated by CRL4^Cdt2^ through common molecular mechanisms by which it controls its other substrates, and speaks to the significance of Pol δ3 as a participant in DNA replication. The cell cycle variations in p12, p21 and Cdt1, broadly follow comparable time courses consistent with their regulation by CRL4^Cdt2^. This has been demonstrated at the single cell level by laser scanning cytometry, coupled with analysis of DNA replication by 5-Ethynyl-2’-deoxyuridine (EdU) labeling [118,119]. 

### 2.8. Mechanism and Characteristics of Okazaki Fragment Processing by Pol δ4 and Pol δ3 

Discontinuous DNA synthesis at the lagging strand in eukaryotes involves the synthesis of Okazaki fragments of ca. 200 nucleotides. The process of Okazaki fragment maturation is essentially one where they are joined to the growing lagging strand. The key elements of this process have been characterized by biochemical reconstitution and genetic studies [33,120]. In yeast, Pol δ has been shown to have properties that are conducive to a role in Okazaki fragment processing. One of these is its propensity to idle at a nick, thereby allowing DNA ligase action [121]. On encounter with a 5′ end of the previous Okazaki fragment, Pol δ will advance several nucleotides because of fraying of the primer end and strand displacement, creating short flaps. These short flaps are then cleaved by Flap endonuclease 1 (Fen1) [120,122], so that the primer ends are removed. This process is termed the short flap pathway, and the products are predominantly mononucleotides and short oligonucleotides of 2–10 nucleotides (nt). However, Fen1 does not cleave longer flaps, and the accumulation of longer flaps acts as a barrier to Okazaki fragment maturation and is a potential source of genomic instability. While yeast Pol δ is able to idle at a nick, it does possess a significant ability for strand displacement [123], so that creation of long flaps can take place. A second pathway, the “long flap pathway”, cleaves these long flaps via the actions of Pif1 helicase and Dna2 to a length that allows their removal by Fen1 [120,124]. The final step is ligation of the nick by DNA ligase I [125]. This can be contrasted to the situation in prokaryotes, where the removal of the primers is performed by a nick translation mechanism in which Pol I both extends the primer end and excises single nucleotides from the 5′ end of the prior Okazaki fragment by virtue of its 5′ to 3′ exonuclease activity [126]. 

The behavior of human Pol δ4 and Pol δ3 in the component and complete reactions of Okazaki fragment processing were compared in a reconstituted system [59,127]. The key observations were that Pol δ4 is proficient in strand displacement, and performs Okazaki fragment processing in a manner similar to that of yeast Pol δ in combination with Fen1. The spectrum of flap sizes ranges from 1 to 8 nt, but is dependent on Fen1 concentration. With increasing Fen1 the product spectrum is shifted to 1–3 nt, with the mononucleotide prevailing. Pol δ3 does not perform strand displacement. With Fen1 and Pol δ3 the primary products are single nucleotides and a smaller amount of di- and trinucleotides. The rate and nature of product formation distribution is relatively unaffected by Fen1 concentration, supporting the proposal that mammalian Okazaki processes might involve a PCNA/Pol δ3/Fen1 complex [127], in analogy to that which has been demonstrated in the Archaeal system [128].

The question then arises, why do we need two Pol δ forms for lagging strand synthesis? The answer may lie in the complex nature of genomic DNA. It is possible that there are template regions that Pol δ4 is more capable than Pol δ3 of traversing. In this view, we would assign Pol δ3 as the primary agent for Okazaki fragment synthesis and processing. The preference for the use of Pol δ3 and a nick translation mode of Okazaki fragment processing lies in the avoidance of the generation of long flaps. Pol δ4 could also be used in Okazaki fragment processing, under circumstances discussed below (see Section 3.4).

The large number of Okazaki fragments that needs to be generated during synthesis of the human genome requires that the process be highly efficient, and the tendency of Pol δ to frequently dissociate is compatible with the need for rapid recycling of Pol δ (see Section 1.2). The properties that Pol δ displays in Okazaki fragment processing are similar to those needed for gap filling in DNA repair in terms of the length of DNA synthesis that is required [127]. The role of PCNA may hold more importance in this context as a platform for coordinating the reactions of Pol δ, Fen1 and DNA ligase I than its role as a processivity factor. The kinetic constants for Pol δ4 and Pol δ3 [75] provide for an estimate or a prediction of their processivity, based on the ratio of *k*_pol_ to *k*_off_ which can be approximated by *k*_cat_ [129]. These provide values of 350 and 106 nt for Pol δ4 and Pol δ3 respectively. In contrast, yeast Pol δ is able to sustain DNA synthesis in a strictly processive manner to at least 5 kb [130].

The findings that yeast Pol δ is adapted for Okazaki fragment maturation have led to extensive studies that support a division of labor between Pol δ and Pol ε at the replication fork, where Pol δ is the lagging strand polymerase and Pol ε is the leading strand polymerase. Much of the evidence for a division of labor is based on several genetic studies using mutant polymerases that allow discrimination between leading and lagging strand DNA synthesis (reviewed in [131]). How human Pol δ fits into this concept must now also take into account the presence of Pol δ4 and Pol δ3, although their properties suggest that they are more adapted to lagging than leading strand synthesis (see Section 3.3 and Section 3.4 below for further discussion). 

A controlled balance between Pol δ4 and Pol δ3 appears to be required in vivo for genomic stability. Reduced expression of the *POLD4* gene has been associated with lung cancer and a poor prognosis for certain lung cancer patients [132,133]. siRNA suppression of p12 in cultured cells was found to lead to cell cycle delay, and an elevated frequency of chromosomal aberrations [132,133]. 

## 3. Role of the Pol δ Binding Protein PDIP46/Poldip3 in DNA Replication and Repair

There has been a search for accessory or auxiliary proteins that could modulate Pol δ activity since its initial discovery. Two novel Pol δ interacting proteins of previously unknown functions, PDIP46 (Poldip3) and PDIP38 (Poldip2), were discovered by yeast two hybrid screening with the p50 subunit of Pol δ as the bait [134]. An independent study identified tumor necrosis factor α and interleukin 6 inducible protein (TNFAIP1) as a Pol δ binding protein (PDIP1/Poldip1) [135]. All three Pol δ interacting proteins share in common the abilities to bind to the p50 subunit and PCNA. The functions of Poldip1, PDIP38/Poldip2 and PDIP46/Poldip3 in relation to Pol δ have proven to be enigmatic, and they appear to be multifunctional proteins. 

**Figure 4:**
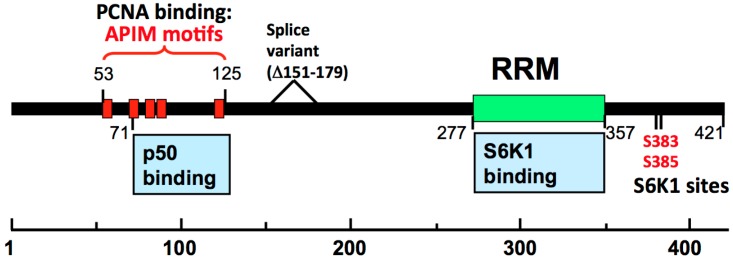
Domain map of PDIP46/SKAR.

PDIP46 was re-discovered as S6K1 Aly/REF-like target (SKAR) [136]. SKAR possesses a RRM (Figure 4) with strong homology to the Aly/REF RNA binding proteins. The latter are involved in coupling transcription with pre-mRNA splicing and mRNA export [136]. S6K1 (ribosomal protein S6 kinase-1) lies downstream of the mTOR and PI3K signaling pathways that regulate cell growth and proliferation through nutrient, energy and mitogenic signals [137,138]. SKAR is a nuclear protein, and is also present in the nuclear speckles and the EJC (exon junction complex) where it acts to enhance translational efficiency [139,140,141]. Activation of S6K1 through the mTOR and PI3K signaling pathways leads to phosphorylation of PDIP46 at S383/S385. This phosphorylation is required for the binding of activated (phosphorylated) S6K1 binding to PDIP46 (Figure 4). This leads to their recruitment to the spliceosomes where S6K1 regulates translational efficiency [136,141]. siRNA depletion of S6K1 leads to smaller cell size [138], and this effect is also produced by siRNA depletion of SKAR and 4EBP1/eIF4E [142]. Thus, PDIP46 serves to translocate activated S6K1 to the spliceosome, subsequent to the activation of the mTOR pathway. Whether PDIP46, which possesses an Aly/REF type of RNA binding domain, also independently affects mRNA metabolism is unknown. However, PDIP46 is also a binding partner of enhancer of rudimentary homolog (ERH) [143]. ERH is a transcriptional regulator that affects the expression of a number of genes in the cell cycle as well as genes involved in DNA damage including ATR, and genes involved in DNA replication [144,145,146]. 

Recently, PDIP46 has been shown to have a role in the activation of Pol δ activity [56]. These effects are reviewed below, and point to an important role for modulating Pol δ activity. Its functions in this regard are consistent with its other roles in growth regulation studied as the SKAR protein. Thus, PDIP46 appears to be a multifunctional protein.

### 3.1. Mapping of the Interaction Sites between PDIP46 and Pol δ /PCNA Reveals that These Are Located in a Region Separate from Those Involved in S6K1 Binding

The interaction sites of PDIP46 with PCNA and the p50 subunit were mapped to residues 71–125 and that for PCNA between residues 53 and 125 (Figure 4). The PCNA binding of PDIP46 is due to its possession of a cluster of five APIM motifs [56]. The APIM (AlkB homologue 2 PCNA-Interacting Motif) is a novel PCNA binding motif that was first identified in the human DNA repair enzyme oxidative demethylase AlkB Homologue 2 (ABH2) [147]. The APIM consists of five residues with the consensus sequence [KR]-[FYW]-[LIVA]-[LIVA]-[KR]. Seven other proteins have been shown to have functional APIM motifs. These include Topo IIα [147], the NER protein XPA [148] and the F-box helicase FBH1 [149] that is involved in homologous recombination. The APIM motif binds to the same regions of PCNA as the PIP-box [150]. The separation of the locations of the PCNA/Pol δ binding regions from the RRM/S6K1 binding domain in the C-terminus (Figure 4) is consistent with the possibility that PDIP46 is a bi-functional protein whose two functions are harbored in two separate structural domains [56]. 

### 3.2. Evidence that PDIP46 Is Associated with Pol δ In Vivo

There is supportive evidence that PDIP46 interacts with Pol δ in a cellular context. This has been demonstrated by their co-immunoprecipitation and co-elution during affinity chromatography on immobilized anti-p125 monoclonal antibody. ChIP analysis with antibody against the p125 subunit showed that PDIP46 was present together with two components of the mammalian replisome [56]. These are Mcm2, a component of the Cdc45-MCM-GINS (CMG) helicase [151], and Ctf4, which associates with CMG [152,153]. Thus, the ChIP data supports the idea that PDIP46 is associated with chromatin at or near the replisome.

### 3.3. PDIP46 Is a Potent Activator of Pol δ

All PCNA binding proteins possess the ability to compete with Pol δ for PCNA, and therefore can inhibit Pol δ in activity assays at sufficiently high concentrations. This was found to be the case for PDIP46 [56] and PDIP38 [154], when assayed using poly(dA)/oligo(dT) as the substrate. More recently, the effects of PDIP46 on Pol δ activity were examined in the M13 assays in which PCNA is pre-loaded onto the primer end with RFC. This assay is more reflective of DNA synthesis in vivo than the standard assay using poly(A)/oligo(dT) as the substrate (see Section 1.2). PDIP46 was revealed to be a remarkably potent activator (ca. 10 fold) of Pol δ4 in the synthesis of the 7 kb M13 DNA, with an apparent *K*_d_ of ca. 34 nM [56]. The mechanisms for this activation could be due to several causes. These include an increase in processivity, possibly because PDIP46 may stabilize Pol δ binding to PCNA by a bridging interaction, as well as by a direct activation that involves alteration of the kinetic properties of Pol δ4.

The effects of PDIP46 were examined on model oligonucleotide templates [127] in assays that examined primer extension and strand displacement in order to gain insights into its mechanism(s) of action [56]. In the absence of PCNA, Pol δ4 behaves in a distributive fashion, and PDIP46 clearly stimulates this activity. These results demonstrate that PDIP46 exerts a direct effect on Pol δ4. In the presence of PCNA, the reactions are much faster but it was nevertheless observed that Pol δ4 activity is stimulated. Pol δ3 activity was much less affected than Pol δ4 activity. PDIP46 also stimulated the strand displacement activity of Pol δ4 using model templates with a blocking oligonucleotide, both in the absence and presence of PCNA. Little or no effects were observed on Pol δ3, which does not exhibit strand displacement activity [127]. 

**Figure 5:**
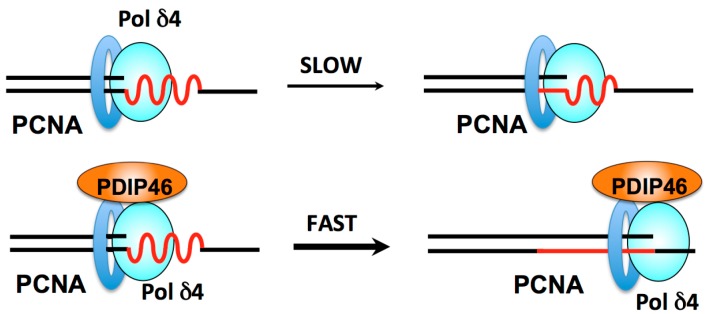
PDIP46 activates the extension of primers across regions of secondary structure in the template (shown in red).

Next, the effects of PDIP46 on an oligonucleotide substrate with a hairpin/stem-loop (16-nt stem, 8-nt loop) were examined. PDIP46 stimulated Pol δ4 synthesis through the stem-loop by ca. four-fold. While these effects are smaller than those observed with the M13 template, they explain the greatly increased rate of accumulation of full-length products by Pol δ4 on the M13 template in the presence of PDIP46. The M13 template may have many regions of secondary structures. Thus, there would be a cumulative effect on overall rates of Pol δ4 synthesis in the presence of PDIP46 [56]. PDIP46 could act by stabilization of the Pol δ4/PCNA/DNA complex by a bridging interaction (Figure 5), as well as by a direct activation that involves alteration of the kinetic properties of Pol δ4. The effects of PDIP46 on Pol δ4 are highly relevant in the context of chromosomal replication (Section 3.4.2 below). 

These studies also highlight the connection between strand displacement and the ability of Pol δ to synthesize through a hairpin structure. Once Pol δ encounters the hairpin, further synthesis through the stem portion of the hairpin is analogous to the process of strand displacement [56]. Thus, it is not surprising that Pol δ3 exhibits minimal activity with the hairpin substrate as it does not perform strand displacement activity. 

Mutations of PDIP46 in which all of the APIM motifs are mutated abolished the effects of PDIP46 on Pol δ4, validating the assignment of PCNA binding to this region. Deletion of the RRM has no effect on the activation of Pol δ4 by PDIP46, so that PDIP46 appears to have two independent functional domains [56]. 

These studies are the first to document the effects of PDIP46 on Pol δ4, and obviously raise many more questions regarding its mechanism of action. In particular, kinetic studies are needed to establish whether PDIP46 has any effect on the intrinsic catalytic properties of Pol δ4. Such effects could also involve alterations in fidelity. In addition, characterization of the range of complexity of secondary structures in which PDIP46 can act to facilitate Pol δ4 bypass synthesis is important in understanding the extent to which its functions could facilitate Pol δ4 bypass synthesis.

### 3.4. Future Horizons: Accommodating Two Forms of Pol δ and PDIP46 at the Replication Fork

Current models for the respective roles of Pol δ and Pol δ at the replication fork are based on both biochemical and genetic approaches in yeast. Several studies [155,156,157] using error-prone Pol δ and Pol ε support a model where Pol δ and Pol ε function mainly as lagging and leading strand polymerases, respectively (reviewed in [131]). By contrast, it has been argued that genetic approaches also support a model where Pol δ has a major role on both forks [158]. In the case of human DNA replication, the differences in subunit structure and properties between yeast Pol δ and human Pol δ have to be taken into account, in particular the existence of two forms of human Pol δ as well as of PDIP46, which selectively acts on Pol δ4. In the following sections we propose models for their roles in lagging and leading strands. 

#### 3.4.1. Roles of Pol δ3, Pol δ4 and PDIP46 in Lagging Strand Synthesis

**Figure 6:**
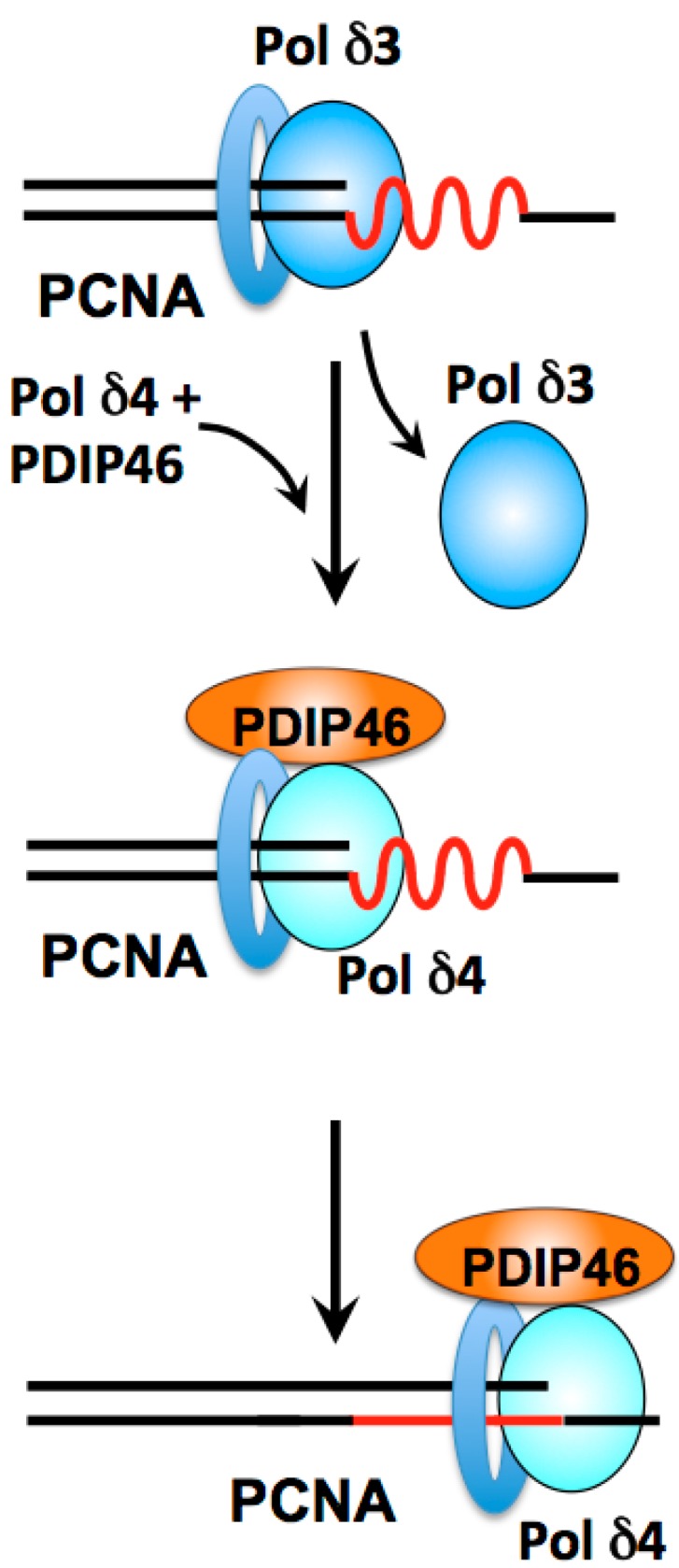
Pol δ3 and Pol δ4/PDIP46 in lagging strand synthesis. Template regions of secondary structure (red) that pose impediments to Pol δ3 leads to dissociation and triggers a polymerase switch to Pol δ4/PDIP46.

Biochemical and reconstitution studies have provided strong arguments for an adaptation of Pol δ for Okazaki fragment synthesis and processing [59,127]. In the human system, we have two forms of Pol δ; how do these fit into our current views of the replication fork? While both Pol δ3 and Pol δ4 are capable of Okazaki fragment processing in vitro, Pol δ3 exhibits the more desirable characteristics of acting through a nick translation mode that avoids the generation of long flaps [59,127]. The model we propose is that they are used interchangeably during Okazaki fragment synthesis. This model is based on studies reviewed above in Section 2.8. In this model (Figure 6) Pol δ3 is the default lagging strand polymerase. When regions of secondary structure which act as barriers to Pol δ3 are encountered [56], Pol δ4 is switched with Pol δ3, together with PDIP46 (Figure 6). 

There are regions of varying template complexity in chromatin that include simple hairpins, microsatellite regions [159] that contain CFS (common fragile sites), and trinucleotide repeats [160]. These pose potential barriers to the replicative polymerases. There has been a broadening view of polymerase usage during normal DNA replication, e.g., the utilization of translesion polymerases, notably Pol κ [161,162] and Pol η in chromosomal DNA replication to augment the functions of replicative polymerases [88,163,164,165]. Future characterization of the range of complexity of secondary structures for which PDIP46 may act to facilitate Pol δ4 bypass synthesis is important to understanding its functions. 

#### 3.4.2. Roles of Pol δ4 and PDIP46 in Leading Strand Synthesis

It is generally accepted that a leading strand polymerase should have high processivity. The loss of Pol ε function in *S. cerevisiae* is nonlethal, indicating that yeast Pol δ can act at both leading and lagging strands [34]. As previously noted, Pol δ3 appears to be much less processive than Pol δ4, so that it is an unlikely candidate for a role in leading strand synthesis (Section 1.2 and Section 2.8). Pol δ4 has been shown to be less processive than Pol ε [166], so that it might be considered also to be a poor candidate for leading strand DNA synthesis. However, PDIP46 could augment Pol δ4 function in leading strand synthesis, in analogy to its effects in the M13 assay that reveal a gain in synthesis rate of about an order of magnitude [56]. Thus, PDIP46 could function as an accessory protein to provide for Pol δ4 with the required speed and processivity in leading strand synthesis. 

**Figure 7:**
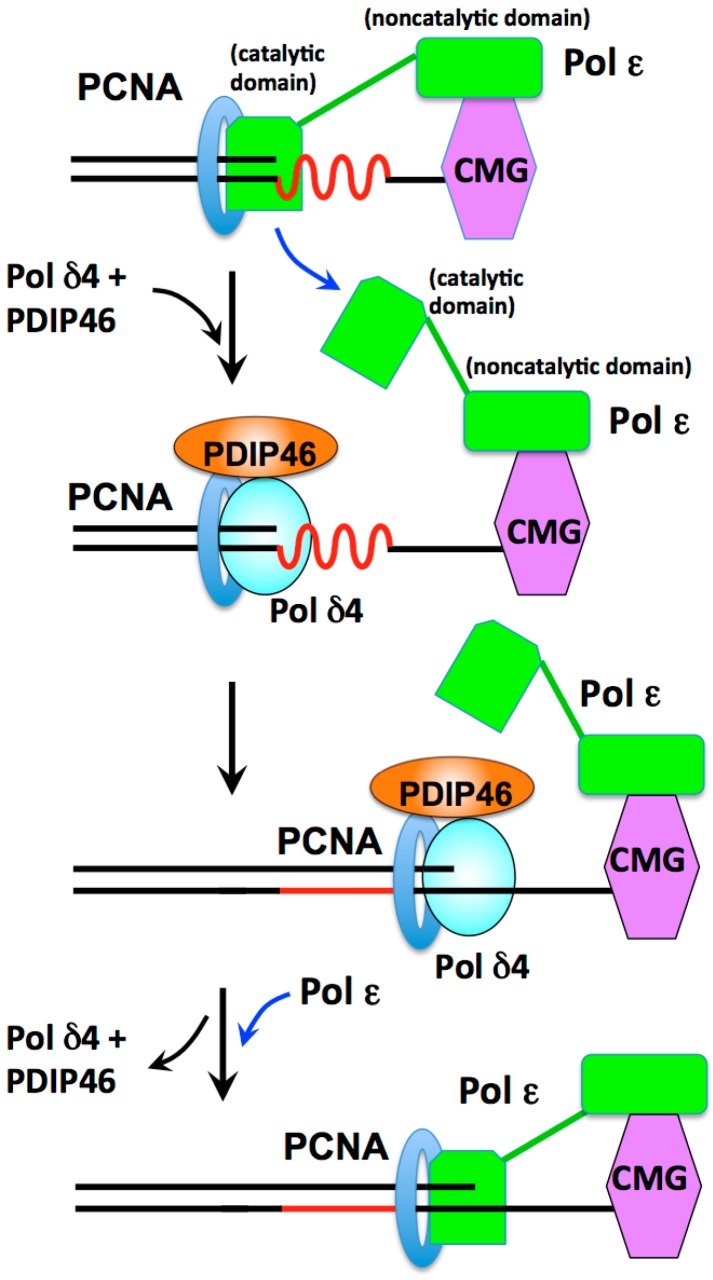
Pol δ4/PDIP46 in leading strand synthesis. Regions of secondary structure that pose impediments to Pol ε (red) lead to disengagement of the catalytic domain of Pol ε. This triggers a polymerase switch to Pol δ4/PDIP46.

In addition to a general role as a leading strand polymerase, Pol δ4/PDIP46 could act in an analogous way as proposed above (Figure 6) in lagging strand synthesis. We propose that Pol ε may stall at regions of secondary structure, and then is switched for Pol δ4/PDIP46 (Figure 7). The ability of Pol ε to bypass complex template DNA regions has not been extensively studied. However, Pol ε exhibits only minimal strand displacement activity, like Pol δ3, and has been shown to be unable to perform strand displacement [167]. Thus, it might be predicted that Pol ε, like Pol δ3 [56], could potentially stall at regions of template secondary structures. This model, like that for the lagging strand, views Pol δ4 (with PDIP46) as functioning as a specialized polymerase to deal with regions of secondary structure that stall Pol ε. 

The model shown in Figure 7 incorporates recent structural and functional studies of the yeast replisome from the Diffley laboratory [168,169,170,171]. These studies show that the catalytic domain of Pol ε is flexibly attached to its non-catalytic domain (which is engaged in complex with the CMG helicase). The catalytic domain adopts two conformations: it is proposed that in one conformation the catalytic domain is actively engaged with the DNA and in the other one it is disengaged [168,169]. In the context of the human replisome, we envisage that encounter with replication blocks stalls Pol ε, leading to the disengagement of the catalytic domain, followed by a switch to Pol δ4/PDIP46 which performs the bypass synthesis (Figure 7). When Pol δ4/PDIP46 encounters the CMG helicase, they dissociate and the Pol ε catalytic subunit re-engages the primer terminus. 

The concept that the Pol ε catalytic domain can disengage from the DNA while remaining an integral part of the CMG-helicase leads to a paradigm shift in our thinking of the replisome [168,169]. Thus, where previously disengagement of Pol ε would require a physical uncoupling, this is no longer the case. It was proposed that Pol ε can disengage in response to replication stress, a situation that entertains the possibility of polymerase switching [168,169]. This is the situation where Pol δ4/PDIP46 could come into play in the model shown in Figure 7. 

The studies of the yeast replisome have in fact produced evidence for a switch between Pol δ and Pol ε, and a mechanism for dealing with uncoupling events. Reconstitution of the initiation of DNA synthesis supports a model where Pol α forms the primer; this is extended by Pol δ which then “catches up” with the replisome to hand off the 3′ end of the leading strand to Pol ε in the advancing and uncoupled CMG-Pol ε replisome [168,169]. This is essentially a relay of polymerase handoffs where Pol δ has the anchor role of bringing the growing primer terminus to the CMG-Pol ε. This function of Pol δ in the yeast replisome provides a mechanism to deal with uncoupling events in a more general context, as during replication stress [168,169]. In a sense, the view of Pol ε being able to disengage from the primer end without dissociating from the CMG helicase allows it to have its cake and eat it. Other studies have also shown that Pol δ dissociates once it encounters the CMG-helicase by a collision release mechanism; this was taken as a means of selection against Pol δ at the leading strand [172,173]. However, it is noted that this would be dependent on the frequency of disengagement of Pol ε.

Once the possibility of switching of Pol δ and Pol ε is admitted, arguments against the participation of Pol δ in leading strand synthesis based on our previous understanding of the leading strand replisome as a tightly complexed structure are weakened. Polymerase switching involving Pol ε suggests a far more dynamic replisome. Recent kinetic studies have indicated that human Pol δ dissociates much more frequently than was previously thought [174]. The bacterial replisome is the prototype of a fixed structural assembly of both leading and lagging strand polymerases with the clamp loader. However, recent studies indicate that there is a frequent exchange of the bacterial polymerase during replication [175]. In the case of Pol ε, a similar situation could exist in terms of disengagement, such that uncoupling might be more frequent than expected even in the absence of replication stress. 

These ideas have significant bearing on the participation of Pol δ4 in leading strand synthesis. There are no comparable studies that bear on the distribution of labor between Pol δ and Pol ε for the replication of the far larger and more complex genome in human cells. However, it is noted that replication of the SV40 genome in reconstituted systems can be achieved with Pol α and Pol δ [176,177]. One study using cross-linking and immunoprecipitation approaches, as well as immuno-electron microscopy, has provided evidence that Pol δ and Pol ε could be functioning independently in early and late S phase in the human system [178]. Taking into account recent views on the interplay between Pol δ and Pol ε during leading strand synthesis in yeast discussed above, it would appear that Pol δ may participate more extensively in leading strand synthesis than previously recognized. 

There is a broader significance to the discovery that the catalytic domain of Pol ε is able to “switch” away from the DNA. It was proposed that Pol ε could also disengage from the DNA during replication stress [168,169]. Replication stress, broadly defined as encounter with replication barriers due to template lesions or complex DNA structures might be addressed by similar mechanisms to that which are well established in relation to Pol δ [88,179,180] and are based on the switching of specialized polymerases such as the TLS polymerases. The ability of Pol ε to disengage could also be involved in replication restart mechanisms that involve re-priming by PrimPol [181]. In the case of re-priming, the ability of Pol ε to disengage could also open the possibility of Pol α being able to re-prime, recapitulating the process that occurs during initiation [168,169]. These possibilities point to a convergence and a more unified view of mechanisms that deal with replication stress at both the leading and lagging strands of the replication fork. 

There is still much to learn about the functions of PDIP46 in DNA replication. In addition to further biochemical analyses of the mechanisms by which PDIP46 affects Pol δ4, are questions as to whether PDIP46 functions are regulated by the mTOR pathway or by DNA damage signaling pathways. The functions of PDIP46 as SKAR indicates that it is a bifunctional protein, pointing to a need for structural studies, as well as careful dissection of the two functions, to allow design of appropriate mutations that selectively target its effects on Pol δ. The proposed roles of PDIP46 in DNA replication would be expected to yield phenotypes in a cellular context when its functions are disabled that reflect disturbances in DNA replication and genomic stability. Recent studies of genetic alterations in cells derived from high-risk neuroblastoma tissues have identified a group of genes whose alterations in copy number resulted in high tumorigenic capacity. The PDIP46 gene (*POLDIP3*) was one of six genes whose lowered expression was correlated with decreased overall and relapse free survival in a cohort of 88 patients [182]. Along with this, immunohistochemical tissue staining reveals a pattern of lowered expression in over 20 of the most common cancers (Human Protein Atlas, [183]). The catalogue of somatic mutations in cancer (COSMIC) database also showed that significant up- or down-regulation of POLDIP3 as being associated with various cancers as well as a number of mutations [184]. This provides some evidence that PDIP46 function is involved in maintenance of genomic stability.

## 4. PDIP38/Poldip2: A Multi-Faceted Protein

Recombinant mature PDIP38 at high concentrations (micromolar) inhibit Pol δ activity, an effect likely due to competition for PCNA that is unlikely to be physiologically relevant [154]. Thus despite its binding to Pol δ, the effects of PDIP38 on Pol δ, if any, are as yet not well defined. Studies reviewed below implicate PDIP38 in a number of cellular processes that are diverse, and further complicated by its localization to multiple subcellular organelles and structures, as well as its association with multiple protein partners including a transmembrane enzyme. 

### 4.1. PDIP38 Is a Mitochondrial Protein with Multiple Subcellular Localizations

Analysis of the subcellular localization of PDIP38 revealed that it is primarily a mitochondrial protein. PDIP38 possesses a mitochondrial targeting site located in the N-terminal 30 amino acid residues, and cleavage sites [185] for mitochondrial processing peptidase and mitochondrial intermediate peptidase [154]. The N-terminal 50 residues are efficiently removed to yield a 38 kDa protein rather than the expected 42 kDa precursor. Cell fractionation experiments indicated that the bulk of the PDIP38 in cells was in a mitochondrial pellet, and resistant to proteinase K digestion until the membranes were solubilized with Triton X-100; a smaller amount was present in the nuclear fraction. Immunofluorescence studies of endogenous PDIP38 as well as of ectopically expressed C-terminally-tagged EGFP constructs showed that they are localized to the mitochondria [154]. Similar fractionation and immunofluorescence studies in two other studies confirmed these findings with the further indication that PDIP38 is present in the mitochondria matrix [186,187]. PDIP38 was found to associate with mitochondrial single stranded binding protein (mt SSB) and with the mitochondrial DNA nucleoid/mitochromosome [186,187]. The functions of PDIP38 in mitochondria are still unclear; in addition to potential effects on mitochondrial DNA replication, its depletion affects mitochondrial morphology [187], raising a question of whether the effects of its depletion also impacts mitochondrial energy metabolism. 

There are conflicting reports on the subcellular localization of PDIP38. PDIP38 was found to be an interacting protein for CEACAM1, a cell adhesion receptor [188]. Analysis of its subcellular localization using peptide directed antibodies showed that the bulk of the PDIP38 is present in the cytoplasm, but does not co-localize with mitochondrial markers, a result contradictory to the studies described above. The basis of this difference regarding mitochondrial localization from those reported above [154] are unknown, although this could be due to differences in the antibodies used or the fixing of the cells. However, these studies did show significant evidence for PDIP38 in the nuclei. PDIP38 was dynamically localized to the cell surface membranes and the nuclei under influence of CEACAM1 [188]. Further analysis showed that PDIP38 is localized to the mitotic spindle. siRNA depletion of PDIP38 or microinjection of PDIP38 antibodies was associated with the appearance of aberrant spindle formation, chromosome segregation, as well as multinucleate cells [189]. 

### 4.2. Interaction of PDIP38/Poldip2 with Pol η and Other TLS Polymerases: Involvement of PDIP38 in the DNA Damage Tolerance Pathway

PDIP38 was found to interact directly with Pol η by a yeast two-hybrid screen with Pol η as the bait. Depletion of Pol η, PDIP38, or both, led to similar degrees of increased sensitivity to UV in cell survival assays. This suggested that PDIP38 plays an integral role in Pol η function [190]. The molecular mechanisms of the connections between PDIP38 and Pol η remain to be elucidated, but it has been suggested that PDIP38 might be a mediator in the switching process between Pol δ and Pol η [190]. In this context, PDIP38 might act to facilitate the recruitment of Pol η to Pol δ stalled at UV lesions. PDIP38 was also found to bind to the specialized polymerases Rev1 and Pol ξ (through interaction with the Rev7 subunit) [190]. These findings indicate that PDIP38 might be involved in the functions of other specialized DNA polymerases. The functional effects of PDIP38 on Rev1 and Pol ξ have not been reported.

Analysis of the effects of PDIP38 on five different DNA polymerases (Pols δ, η, ι, λ and β) showed that only the activities of Pol δ, Pol η and Pol λ were affected, consistent with the finding that Pol λ also physically interacts with PDIP38 [191]. PDIP38 (as the full-length protein) stimulated the processivity and catalytic activities of Pol η and Pol λ at low nanomolar concentrations on oligonucleotide templates containing lesions that included 8-oxoG, abasic sites and thymine-thymine dimers [191]. Additionally, the error-free bypass of 8-oxoG was increased, and a stimulatory effect on Pol δ was also found. Pol λ [192,193,194] participates in base excision repair of oxidative damage of guanine bases, as well as in a form of nonhomologous end joining repair of DSBs [193,195,196,197]. It was also demonstrated that depletion of PDIP38 led to an increase in the sensitivity of cultured cells to oxidizing agents [191]. Recently, a sixth polymerase was added to the list of PDIP38 binding proteins, this being PrimPol [198]. PrimPol is a member of the archaeo-eukaryotic primase (AEP) superfamily and exhibits primase, polymerase as well as translesion polymerase activities, and has emerged as having the ability to reprime DNA replication at sites of replication stress [181]. The effects of PDIP38 on PrimPol mirror those found for Pol η and Pol λ, viz., activation, increased processivity and fidelity for bypass of 8-oxoG. Depletion of either PDIP38 or PrimPol (or both) gave rise to replication defects (decrease in replication fork rates) in response to UV damage, suggesting that they are linked in the same pathway in vivo [198].

That PDIP38 is capable of interactions with a diverse group of polymerases raises interesting questions as to how this is achieved. Two similar short amino-acid sequences that are involved in PDIP38 binding were identified in Pol η [190] and in PrimPol [198]. An N-terminal sequence within the mitochondrial targeting sequence of PDIP38 was found to be a binding region for PrimPol. Full length PDIP38, but not the processed form, was able to activate PrimPol and Pol η [198]. These findings raise the question of whether levels of the unprocessed form in the nuclei would be sufficient to achieve functional concentrations in vivo, as most of the cellular PDIP38 is in the processed form [154].

The broad versatility of PDIP38 in the regulation of these polymerases, all of which are involved in the relief of replication stress, makes the elucidation of its structure and the location of its interaction sites an important goal. Furthermore, the apparently wide reach of PDIP38 in modulating activities of polymerases involved in translesion synthesis and relief of replicative stress indicates that it is likely to be under the control of the DNA damage tolerance regulatory pathways, notably those under the apical ATR kinase. 

### 4.3. PDIP38 Responds to Genotoxic and Transcriptional Stress by Translocation to the Spliceosomes/Nuclear Speckles and Is Involved in Regulation of the Alternative Splicing of Mdm2

The potential involvement of PDIP38 in Pol η function suggests that it should be recruited to UV damage foci [190]. Using the technique of UV exposure through UV-opaque polycarbonate filters with 5 or 10 µm pores to create local areas of irradiation [84], it was observed that PDIP38 was not recruited to these DNA damage foci, in contrast to Pol η and PCNA [199]. This finding does not negate the proposal that PDIP38 is involved in Pol η recruitment [190], since the mechanism is unknown and might involve a transient association of PDIP38 to DNA damage sites. 

Examination of the nuclear localization of PDIP38 showed that it was nevertheless recruited to nuclear foci in response to UV. These nuclear foci were identified as spliceosomes (nuclear speckles), which are associated with transcription and mRNA splicing processes [200]. Treatment with UV increased the number of cells with visible PDIP38 foci, as well as the number of foci per cell. Thus, the translocation of PDIP38 to the spliceosomes is a novel DNA damage response [199]. In addition to genotoxic stress, transcriptional stress induced by α-amanitin also led to translocation of PDIP38 to the nuclear speckles [199]. Interestingly, the human DNA glycosylase hOGG1 is also translocated to the nuclear speckles under the influence of UVA [201].

The translocation of PDIP38 in response to UV-damage raises the question of its functions in the spliceosomes/nuclear speckles, which are associated with transcription and mRNA splicing. There are a number of genes whose alternative splicing is altered under genotoxic stress [202,203]. One of the more extensively studied of these genes is mouse double minute 2 (*MDM2*) [202,204]. Mdm2 is an E3 ubiquitin ligase that is a negative regulator of p53 [202,205]. Various genotoxic agents, e.g., UV, camptothecin, doxorubicin and cisplatin, lead to skipping of as many as eight exons, resulting in disruption of Mdm2 function and of p53 regulation [205]. Alternative spliced variants of Mdm2 also can exhibit growth regulatory properties independent of p53 and induce tumorigenesis [206,207,208,209]. 

Analysis of the UV induced Mdm2 splice variants in A549 cells showed that this was dependent on PDIP38, as their levels were suppressed in PDIP38 depleted cells [199]. While the extent and mechanisms that underlie the basis for the requirement for PDIP38 in Mdm2 alternative splicing are unknown, it may be another example of the interplay or crosstalk between DNA damage/repair processes and RNA transcription/splicing in the maintenance of genomic stability and cell survival [203,210,211]. This crosstalk has largely focused on RNA binding proteins, but also on the involvement of DNA damage response proteins in regulating splicing factors [210]. The effects of PDIP38 in modulating the splicing of Mdm2, a key regulator of p53, falls into this category, and may represent one of its important functions. 

### 4.4. PDIP38 Binds to p22phox and Regulates the Activity of the Nox4/p22phox NADPH Oxidase

Nox4 (NADPH oxidase 4) is one of seven transmembrane NADPH oxidases that generate reactive oxygen species (ROS): superoxide and H_2_O_2_ [212,213,214,215]. The generation of ROS by the NOX enzymes occurs physiologically in response to various stimuli; these ROS act on signal transduction pathways [212,213,216,217]. Nox4 is widely distributed in tissues, with the highest levels in kidney [213]. Biochemical analysis of partially purified membrane free preparations of Nox4 revealed that Nox4 has a high K_m_ for O_2_, and functions as an oxygen sensor, in that its activity responds to the physiological pO_2_ [218]. These studies also demonstrated that the Nox4 reaction generates H_2_O_2_ as the primary product with a smaller amount of superoxide [218]. This response to pO_2_ has relevance to the proposed role for Nox4 as an oxygen sensor that produces H_2_O_2_ as a signaling molecule [218]. Four of the Nox enzymes including Nox4 are associated with p22phox, which acts as a subunit that interacts with regulatory proteins in response to cellular stimuli [212,219]. However, Nox4 binds p22phox which is required for its activity, and is regarded as being constitutively active [213,215]. 

The role of PDIP38 in regulating Nox4 functions has been extensively studied in the cardiovascular system [215,219]. PDIP38 was found to bind to p22phox and to activate the Nox4/p22phox enzyme in vascular smooth muscle cells [220]. The latter study supports the view that PDIP38 is involved in Nox4 localization, focal adhesion integrity, stress fiber formation, and plays a role in the maintenance of vascular smooth muscle cell cytoskeletal functions. PDIP38 was found to co-localize with p22phox at focal adhesions and stress fibers [220]. Overexpression of PDIP38 in vascular smooth muscle cells increased NADPH oxidase activity several fold in a Nox4 dependent manner [220]. PDIP38 also regulates vascular smooth muscle cell migration by regulating focal adhesion turnover and traction force generation [221]. PDIP38 knockout in mice has shown that it is essential for development, as this led to perinatal lethality [222]. Analysis of aortas from heterozygous mice showed that these exhibited abnormal structures and decreased contraction and compliance that are consistent with a role in vascular function and integrity. Mouse embryonic fibroblasts derived from the knockout mice exhibit defective growth characteristics, alterations in cell cycle progression and expression of cell cycle proteins [223]. The subcellular localizations of Nox4/p22phox and PDIP38 in vascular smooth muscle cells also raises questions regarding how these integrate into the fact that the Nox enzymes are membrane associated proteins [212]. 

### 4.5. Summary

PDIP38 is unusual in that there is evidence for its role in a number of cellular functions, emanating from the discovery of multiple protein partners. In addition to Pol δ and PCNA, PDIP38 interacts with Pol η and other TLS pols. These findings together indicate a role for PDIP38 in regulating translesion synthesis, while its association with Pol δ suggests it may be involved in the mechanisms or regulation of the interchange between the TLS pols and Pol δ. In addition, PDIP38 is likely under regulation from DNA damage signaling pathways and is translocated to the spliceosomes where it affects Mdm2 splicing and thereby p53 regulation. 

Nevertheless, the studies of PDIP38 are still in their early stages, and its multifunctional nature poses significant technical challenges to the use of gene depletion or knockouts either in cells or animals, as these approaches may not allow unambiguous cause and effect relationships. Thus, much further investigation is required to establish how these functions are accomplished at the molecular level, as well as the cellular advantages of the investiture of these functions in a single protein. These require biochemical approaches and, in particular, the elucidation of PDIP38 structure and its complexes with its partners. These could lead to strategies for the use of targeted mutations that could provide the means for isolating cause and effect in gene depletion experiments. 

## Figures and Tables

**Table 1:** Subunit compositions of Pol δ.

Human	p125	p50	p68	p12
*Schizosaccharomyces pombe*	Pol3	Cdc1	Cdc27	Cdm1
*Saccharomyces cerevisiae*	Pol3	Pol31	Pol32	-

## References

[1] Kornberg A. (2000). Ten commandments: Lessons from the enzymology of DNA replication. J. Bacteriol..

[2] Barnes R., Eckert K. (2017). Maintenance of Genome Integrity: How Mammalian Cells Orchestrate Genome Duplication by Coordinating Replicative and Specialized DNA Polymerases. Genes.

[3] Nicolas E., Golemis E.A., Arora S. (2016). POLD1: Central mediator of DNA replication and repair, and implication in cancer and other pathologies. Gene.

[4] Weissbach A. (1977). Eukaryotic DNA polymerases. Annu. Rev. Biochem..

[5] Brutlag D., Kornberg A. (1972). Enzymatic synthesis of deoxyribonucleic acid. 36. A proofreading function for the 3′ leads to 5′ exonuclease activity in deoxyribonucleic acid polymerases. J. Biol. Chem..

[6] Muzyczka N., Poland R.L., Bessman M.J. (1972). Studies on the biochemical basis of spontaneous mutation. I. A comparison of the deoxyribonucleic acid polymerases of mutator, antimutator, and wild type strains of bacteriophage T4. J. Biol. Chem..

[7] Reha-Krantz L.J. (2010). DNA polymerase proofreading: Multiple roles maintain genome stability. Biochim. Biophys. Acta.

[8] Byrnes J.J., Downey K.M., Black V., Esserman L., So A.G., Schultz J., Ahmad F. (1976). Selective Inhibition of the 3′ to 5′ Exonuclease Activity Associated with Mammalian DNA Polymerase δ. Miami Winter Symposium: Cancer Enzymology.

[9] Byrnes J.J., Downey K.M., Black V.L., So A.G. (1976). A new mammalian DNA polymerase with 3′ to 5′ exonuclease activity: DNA polymerase δ. Biochemistry.

[10] Byrnes J.J., Downey K.M., Que B.G., Lee M.Y., Black V.L., So A.G. (1977). Selective inhibition of the 3′ to 5′ exonuclease activity associated with DNA polymerases: A mechanism of mutagenesis. Biochemistry.

[11] Lee M.Y., Byrnes J.J., Downey K.M., So A.G. (1980). Mechanism of inhibition of deoxyribonucleic acid synthesis by 1-beta-D-arabinofuranosyladenosine triphosphate and its potentiation by 6-mercaptopurine ribonucleoside 5′-monophosphate. Biochemistry.

[12] Lee M.Y., Tan C.K., So A.G., Downey K.M. (1980). Purification of deoxyribonucleic acid polymerase δ from calf thymus: Partial characterization of physical properties. Biochemistry.

[13] Lee M.Y., Tan C.K., Downey K.M., So A.G. (1981). Structural and functional properties of calf thymus DNA polymerase δ. Prog. Nucleic Acid Res. Mol. Biol..

[14] Lee M.Y., Tan C.K., Downey K.M., So A.G. (1984). Further studies on calf thymus DNA polymerase δ purified to homogeneity by a new procedure. Biochemistry.

[15] Lee M.Y., Toomey N.L., Wright G.E. (1985). Differential inhibition of human placental DNA polymerases δ and α by BuPdGTP and BuAdATP. Nucleic Acids Res..

[16] Lee M.Y., Toomey N.L. (1987). Human placental DNA polymerase δ: Identification of a 170-kilodalton polypeptide by activity staining and immunoblotting. Biochemistry.

[17] Lee M.Y. (1988). Isolation of multiple forms of DNA polymerase δ: Evidence of proteolytic modification during isolation. Biochemistry.

[18] Lee M.Y., Alejandro R., Toomey N.L. (1989). Immunochemical studies of DNA polymerase δ: Relationships with DNA polymerase α. Arch. Biochem. Biophys..

[19] Lee M.Y., Jiang Y.Q., Zhang S.J., Toomey N.L. (1991). Characterization of human DNA polymerase δ and its immunochemical relationships with DNA polymerase α and epsilon. J. Biol. Chem..

[20] Wong S.W., Syvaoja J., Tan C.K., Downey K.M., So A.G., Linn S., Wang T.S. (1989). DNA polymerases α and δ are immunologically and structurally distinct. J. Biol. Chem..

[21] Zhang J., Chung D.W., Tan C.K., Downey K.M., Davie E.W., So A.G. (1991). Primary structure of the catalytic subunit of calf thymus DNA polymerase δ: Sequence similarities with other DNA polymerases. Biochemistry.

[22] Hao H., Jiang Y., Zhang S.J., Zhang P., Zeng R.X., Lee M.Y. (1992). Structural and functional relationships of human DNA polymerases. Chromosoma.

[23] Yang C.L., Chang L.S., Zhang P., Hao H., Zhu L., Toomey N.L., Lee M.Y. (1992). Molecular cloning of the cDNA for the catalytic subunit of human DNA polymerase δ. Nucleic Acids Res..

[24] Crute J.J., Wahl A.F., Bambara R.A. (1986). Purification and characterization of two new high molecular weight forms of DNA polymerase δ. Biochemistry.

[25] Syvaoja J., Linn S. (1989). Characterization of a large form of DNA polymerase δ from HeLa cells that is insensitive to proliferating cell nuclear antigen. J. Biol. Chem..

[26] Burgers P.M., Bambara R.A., Campbell J.L., Chang L.M., Downey K.M., Hubscher U., Lee M.Y., Linn S.M., So A.G., Spadari S. (1990). Revised nomenclature for eukaryotic DNA polymerases. Eur. J. Biochem..

[27] Pospiech H., Syvaoja J.E. (2003). DNA polymerase epsilon—More than a polymerase. Sci. World J..

[28] Prelich G., Tan C.K., Kostura M., Mathews M.B., So A.G., Downey K.M., Stillman B. (1987). Functional identity of proliferating cell nuclear antigen and a DNA polymerase-δ auxiliary protein. Nature.

[29] Choe K.N., Moldovan G.L. (2017). Forging Ahead through Darkness: PCNA, Still the Principal Conductor at the Replication Fork. Mol. Cell.

[30] Mo J., Liu L., Leon A., Mazloum N., Lee M.Y. (2000). Evidence that DNA polymerase δ isolated by immunoaffinity chromatography exhibits high-molecular weight characteristics and is associated with the KIAA0039 protein and RPA. Biochemistry.

[31] Hughes P., Tratner I., Ducoux M., Piard K., Baldacci G. (1999). Isolation and identification of the third subunit of mammalian DNA polymerase δ by PCNA-affinity chromatography of mouse FM3A cell extracts. Nucleic Acids Res..

[32] Liu L., Mo J., Rodriguez-Belmonte E.M., Lee M.Y. (2000). Identification of a fourth subunit of mammalian DNA polymerase δ. J. Biol. Chem..

[33] Garg P., Burgers P.M. (2005). DNA polymerases that propagate the eukaryotic DNA replication fork. Crit. Rev. Biochem. Mol. Biol..

[34] Gerik K.J., Li X., Pautz A., Burgers P.M. (1998). Characterization of the two small subunits of Saccharomyces cerevisiae DNA polymerase δ. J. Biol. Chem..

[35] Reynolds N., Watt A., Fantes P.A., MacNeill S.A. (1998). Cdm1, the smallest subunit of DNA polymerase d in the fission yeast Schizosaccharomyces pombe, is non-essential for growth and division. Curr. Genet..

[36] Zuo S., Gibbs E., Kelman Z., Wang T.S., O’Donnell M., MacNeill S.A., Hurwitz J. (1997). DNA polymerase δ isolated from Schizosaccharomyces pombe contains five subunits. Proc. Natl. Acad. Sci. USA.

[37] Zuo S., Bermudez V., Zhang G., Kelman Z., Hurwitz J. (2000). Structure and activity associated with multiple forms of Schizosaccharomyces pombe DNA polymerase δ. J. Biol. Chem..

[38] MacNeill S.A., Moreno S., Reynolds N., Nurse P., Fantes P.A. (1996). The fission yeast Cdc1 protein, a homologue of the small subunit of DNA polymerase δ, binds to Pol3 and Cdc27. EMBO J..

[39] Bermudez V.P., MacNeill S.A., Tappin I., Hurwitz J. (2002). The influence of the Cdc27 subunit on the properties of the Schizosaccharomyces pombe DNA polymerase δ. J. Biol. Chem..

[40] Johansson E., Majka J., Burgers P.M. (2001). Structure of DNA polymerase δ from *Saccharomyces cerevisiae*. J. Biol. Chem..

[41] Zhang P., Mo J.Y., Perez A., Leon A., Liu L., Mazloum N., Xu H., Lee M.Y. (1999). Direct interaction of proliferating cell nuclear antigen with the p125 catalytic subunit of mammalian DNA polymerase δ. J. Biol. Chem..

[42] Zhang P., Sun Y., Hsu H., Zhang L., Zhang Y., Lee M.Y. (1998). The interdomain connector loop of human PCNA is involved in a direct interaction with human polymerase δ. J. Biol. Chem..

[43] Roos G., Jiang Y., Landberg G., Nielsen N.H., Zhang P., Lee M.Y. (1996). Determination of the epitope of an inhibitory antibody to proliferating cell nuclear antigen. Exp. Cell Res..

[44] Zhang S.J., Zeng X.R., Zhang P., Toomey N.L., Chuang R.Y., Chang L.S., Lee M.Y. (1995). A conserved region in the amino terminus of DNA polymerase δ is involved in proliferating cell nuclear antigen binding. J. Biol. Chem..

[45] Li H., Xie B., Zhou Y., Rahmeh A., Trusa S., Zhang S., Gao Y., Lee E.Y., Lee M.Y. (2006). Functional roles of p12, the fourth subunit of human DNA polymerase δ. J. Biol. Chem..

[46] Lu X., Tan C.K., Zhou J.Q., You M., Carastro L.M., Downey K.M., So A.G. (2002). Direct interaction of proliferating cell nuclear antigen with the small subunit of DNA polymerase δ. J. Biol. Chem..

[47] Wang Y., Zhang Q., Chen H., Li X., Mai W., Chen K., Zhang S., Lee E.Y., Lee M.Y., Zhou Y. (2011). p50, the Small Subunit of DNA Polymerase Δ, Is Required for Mediation of the Interaction of Polymerase Δ Subassemblies with PCNA. PLoS ONE.

[48] Rahmeh A.A., Zhou Y., Xie B., Li H., Lee E.Y., Lee M.Y. (2012). Phosphorylation of the p68 Subunit of Pol Δ Acts as a Molecular Switch to Regulate Its Interaction with PCNA. Biochemistry.

[49] Podust V.N., Chang L.S., Ott R., Dianov G.L., Fanning E. (2002). Reconstitution of human DNA polymerase δ using recombinant baculoviruses: The p12 subunit potentiates DNA polymerizing activity of the four-subunit enzyme. J. Biol. Chem..

[50] Xie B., Mazloum N., Liu L., Rahmeh A., Li H., Lee M.Y. (2002). Reconstitution and characterization of the human DNA polymerase δ four-subunit holoenzyme. Biochemistry.

[51] Zhou Y., Meng X., Zhang S., Lee E.Y., Lee M.Y. (2012). Characterization of human DNA polymerase δ and its subassemblies reconstituted by expression in the multibac system. PLoS ONE.

[52] Masuda Y., Suzuki M., Piao J., Gu Y., Tsurimoto T., Kamiya K. (2007). Dynamics of human replication factors in the elongation phase of DNA replication. Nucleic Acids Res..

[53] Jiang Y., Zhang S.J., Wu S.M., Lee M.Y. (1995). Immunoaffinity purification of DNA polymerase δ. Arch. Biochem. Biophys..

[54] Podust V.N., Georgaki A., Strack B., Hubscher U. (1992). Calf thymus RF-C as an essential component for DNA polymerase δ and epsilon holoenzymes function. Nucleic Acids Res..

[55] Burgers P.M., Gerik K.J. (1998). Structure and processivity of two forms of *Saccharomyces cerevisiae* DNA polymerase δ. J. Biol. Chem..

[56] Wang X., Zhang S., Zheng R., Yue F., Lin S.H., Rahmeh A.A., Lee E.Y., Zhang Z., Lee M.Y. (2016). PDIP46 (DNA polymerase δ interacting protein 46) is an activating factor for human DNA polymerase δ. Oncotarget.

[57] Gao Y., Zhou Y., Xie B., Zhang S., Rahmeh A., Huang H.S., Lee M.Y., Lee E.Y. (2008). Protein phosphatase-1 is targeted to DNA polymerase δ via an interaction with the p68 subunit. Biochemistry.

[58] Lee M.Y., Zhang S., Lin S.H., Chea J., Wang X., LeRoy C., Wong A., Zhang Z., Lee E.Y. (2012). Regulation of human DNA polymerase Δ in the cellular responses to DNA damage. Environ. Mol. Mutagen..

[59] Lee M.Y., Zhang S., Lin S.H., Wang X., Darzynkiewicz Z., Zhang Z., Lee E.Y. (2014). The tail that wags the dog: p12, the smallest subunit of DNA polymerase δ, is degraded by ubiquitin ligases in response to DNA damage and during cell cycle progression. Cell Cycle.

[60] Zhang S., Zhou Y., Trusa S., Meng X., Lee E.Y., Lee M.Y. (2007). A novel DNA damage response: Rapid degradation of the p12 subunit of DNA polymerase δ. J. Biol. Chem..

[61] Zhang S., Zhao H., Darzynkiewicz Z., Zhou P., Zhang Z., Lee E.Y., Lee M.Y. (2013). A novel function of CRL4Cdt2: Regulation of the subunit structure of DNA polymerase δ in response to DNA damage and during the S phase. J. Biol. Chem..

[62] Kaufmann W.K. (2010). The human intra-S checkpoint response to UVC-induced DNA damage. Carcinogenesis.

[63] Cimprich K.A., Cortez D. (2008). ATR: An essential regulator of genome integrity. Nat. Rev. Mol. Cell Biol..

[64] Flynn R.L., Zou L. (2011). ATR: A master conductor of cellular responses to DNA replication stress. Trends Biochem. Sci..

[65] Conti C., Seiler J.A., Pommier Y. (2007). The mammalian DNA replication elongation checkpoint: Implication of Chk1 and relationship with origin firing as determined by single DNA molecule and single cell analyses. Cell Cycle.

[66] Warren J.J., Forsberg L.J., Beese L.S. (2006). The structural basis for the mutagenicity of *O*(6)-methyl-guanine lesions. Proc. Natl. Acad. Sci. USA.

[67] Hsu G.W., Ober M., Carell T., Beese L.S. (2004). Error-prone replication of oxidatively damaged DNA by a high-fidelity DNA polymerase. Nature.

[68] Meng X., Zhou Y., Zhang S., Lee E.Y., Frick D.N., Lee M.Y. (2009). DNA damage alters DNA polymerase δ to a form that exhibits increased discrimination against modified template bases and mismatched primers. Nucleic Acids Res..

[69] Johnson K.A. (1993). Conformational coupling in DNA polymerase fidelity. Annu. Rev. Biochem..

[70] Kunkel T.A., Bebenek K. (2000). DNA replication fidelity. Annu. Rev. Biochem..

[71] Steitz T.A. (1999). DNA polymerases: Structural diversity and common mechanisms. J. Biol. Chem..

[72] Shamoo Y., Steitz T.A. (1999). Building a replisome from interacting pieces: Sliding clamp complexed to a peptide from DNA polymerase and a polymerase editing complex. Cell.

[73] Donlin M.J., Patel S.S., Johnson K.A. (1991). Kinetic partitioning between the exonuclease and polymerase sites in DNA error correction. Biochemistry.

[74] Khare V., Eckert K.A. (2002). The proofreading 3′-5′ exonuclease activity of DNA polymerases: A kinetic barrier to translesion DNA synthesis. Mutat. Res..

[75] Meng X., Zhou Y., Lee E.Y., Lee M.Y., Frick D.N. (2010). The p12 subunit of human polymerase δ modulates the rate and fidelity of DNA synthesis. Biochemistry.

[76] Ogi T., Limsirichaikul S., Overmeer R.M., Volker M., Takenaka K., Cloney R., Nakazawa Y., Niimi A., Miki Y., Jaspers N.G. (2010). Three DNA polymerases, recruited by different mechanisms, carry out NER repair synthesis in human cells. Mol. Cell.

[77] Terai K., Shibata E., Abbas T., Dutta A. (2013). Degradation of p12 Subunit by CRL4Cdt2 E3 Ligase Inhibits Fork Progression after DNA Damage. J. Biol. Chem..

[78] Bekker-Jensen S., Mailand N. (2010). Assembly and function of DNA double-strand break repair foci in mammalian cells. DNA Repair.

[79] Mailand N., Bekker-Jensen S., Faustrup H., Melander F., Bartek J., Lukas C., Lukas J. (2007). RNF8 ubiquitylates histones at DNA double-strand breaks and promotes assembly of repair proteins. Cell.

[80] Huen M.S., Grant R., Manke I., Minn K., Yu X., Yaffe M.B., Chen J. (2007). RNF8 transduces the DNA-damage signal via histone ubiquitylation and checkpoint protein assembly. Cell.

[81] Harper J.W., Elledge S.J. (2007). The DNA damage response: Ten years after. Mol. Cell.

[82] Yan J., Jetten A.M. (2008). RAP80 and RNF8, key players in the recruitment of repair proteins to DNA damage sites. Cancer Lett..

[83] Sirbu B.M., Cortez D. (2013). DNA damage response: Three levels of DNA repair regulation. Cold Spring Harb. Perspect. Biol..

[84] Chea J., Zhang S., Zhao H., Zhang Z., Lee E.Y., Darzynkiewicz Z., Lee M.Y. (2012). Spatiotemporal recruitment of human DNA polymerase δ to sites of UV damage. Cell Cycle.

[85] Andersen P.L., Xu F., Xiao W. (2008). Eukaryotic DNA damage tolerance and translesion synthesis through covalent modifications of PCNA. Cell Res..

[86] Chen J., Bozza W., Zhuang Z. (2011). Ubiquitination of PCNA and its essential role in eukaryotic translesion synthesis. Cell Biochem. Biophys..

[87] Ghosal G., Chen J. (2013). DNA damage tolerance: A double-edged sword guarding the genome. Transl. Cancer Res..

[88] Sale J.E., Lehmann A.R., Woodgate R. (2012). Y-family DNA polymerases and their role in tolerance of cellular DNA damage. Nat. Rev. Mol. Cell Biol..

[89] Vaisman A., Woodgate R. (2017). Translesion DNA polymerases in eukaryotes: What makes them tick?. Crit. Rev. Biochem. Mol. Biol..

[90] Hoege C., Pfander B., Moldovan G.L., Pyrowolakis G., Jentsch S. (2002). RAD6-dependent DNA repair is linked to modification of PCNA by ubiquitin and SUMO. Nature.

[91] Kannouche P.L., Wing J., Lehmann A.R. (2004). Interaction of human DNA polymerase eta with monoubiquitinated PCNA: A possible mechanism for the polymerase switch in response to DNA damage. Mol. Cell.

[92] Kannouche P.L., Lehmann A.R. (2004). Ubiquitination of PCNA and the polymerase switch in human cells. Cell Cycle.

[93] Zhang Z., Zhang S., Lin S.H., Wang X., Wu L., Lee E.Y., Lee M.Y. (2012). Structure of monoubiquitinated PCNA: Implications for DNA polymerase switching and Okazaki fragment maturation. Cell Cycle.

[94] Murga M., Lecona E., Kamileri I., Diaz M., Lugli N., Sotiriou S.K., Anton M.E., Mendez J., Halazonetis T.D., Fernandez-Capetillo O. (2016). POLD3 Is Haploinsufficient for DNA Replication in Mice. Mol. Cell.

[95] Hirota K., Yoshikiyo K., Guilbaud G., Tsurimoto T., Murai J., Tsuda M., Phillips L.G., Narita T., Nishihara K., Kobayashi K. (2015). The POLD3 subunit of DNA polymerase δ can promote translesion synthesis independently of DNA polymerase ζ. Nucleic Acids Res..

[96] Johnson R.E., Prakash L., Prakash S. (2012). Pol31 and Pol32 subunits of yeast DNA polymerase δ are also essential subunits of DNA polymerase ζ. Proc. Natl. Acad. Sci. USA.

[97] Baranovskiy A.G., Lada A.G., Siebler H., Zhang Y., Pavlov Y.I., Tahirov T.H. (2012). DNA polymerases δ and ζ switching by sharing the accessory subunits of DNA polymerase δ. J. Biol. Chem..

[98] Zhang S., Zhou Y., Sarkeshik A., Yates J.R., Thomson T., Zhang Z., Lee E.Y., Lee M.Y. (2013). Identification of RNF8 as a Ubiquitin Ligase Involved in Targeting the p12 Subunit of DNA Polymerase δ for Degradation in Response to DNA Damage. J. Biol. Chem..

[99] Brzovic P.S., Klevit R.E. (2006). Ubiquitin transfer from the E2 perspective: Why is UbcH5 so promiscuous?. Cell Cycle.

[100] Wang B., Elledge S.J. (2007). Ubc13/Rnf8 ubiquitin ligases control foci formation of the Rap80/Abraxas/Brca1/Brcc36 complex in response to DNA damage. Proc. Natl. Acad. Sci. USA.

[101] Marteijn J.A., Bekker-Jensen S., Mailand N., Lans H., Schwertman P., Gourdin A.M., Dantuma N.P., Lukas J., Vermeulen W. (2009). Nucleotide excision repair-induced H2A ubiquitination is dependent on MDC1 and RNF8 and reveals a universal DNA damage response. J. Cell Biol..

[102] Zhang S., Chea J., Meng X., Zhou Y., Lee E.Y., Lee M.Y. (2008). PCNA is ubiquitinated by RNF8. Cell Cycle.

[103] Abbas T., Dutta A. (2011). CRL4Cdt2: Master coordinator of cell cycle progression and genome stability. Cell Cycle.

[104] Hannah J., Zhou P. (2015). Distinct and overlapping functions of the cullin E3 ligase scaffolding proteins CUL4A and CUL4B. Gene.

[105] Havens C.G., Shobnam N., Guarino E., Centore R.C., Zou L., Kearsey S.E., Walter J.C. (2012). Direct Role for proliferating cell nuclear antigen (PCNA) in substrate recognition by the E3 Ubiquitin ligase CRL4-Cdt2. J. Biol. Chem..

[106] Havens C.G., Walter J.C. (2011). Mechanism of CRL4(Cdt2), a PCNA-dependent E3 ubiquitin ligase. Genes Dev..

[107] Arias E.E., Walter J.C. (2005). Replication-dependent destruction of Cdt1 limits DNA replication to a single round per cell cycle in Xenopus egg extracts. Genes Dev..

[108] Nishitani H., Sugimoto N., Roukos V., Nakanishi Y., Saijo M., Obuse C., Tsurimoto T., Nakayama K.I., Nakayama K., Fujita M. (2006). Two E3 ubiquitin ligases, SCF-Skp2 and DDB1-Cul4, target human Cdt1 for proteolysis. EMBO J..

[109] Higa L.A., Banks D., Wu M., Kobayashi R., Sun H., Zhang H. (2006). L2DTL/CDT2 interacts with the CUL4/DDB1 complex and PCNA and regulates CDT1 proteolysis in response to DNA damage. Cell Cycle.

[110] Soria G., Gottifredi V. (2010). PCNA-coupled p21 degradation after DNA damage: The exception that confirms the rule?. DNA Repair.

[111] Bendjennat M., Boulaire J., Jascur T., Brickner H., Barbier V., Sarasin A., Fotedar A., Fotedar R. (2003). UV irradiation triggers ubiquitin-dependent degradation of p21(WAF1) to promote DNA repair. Cell.

[112] Higa L.A., Mihaylov I.S., Banks D.P., Zheng J., Zhang H. (2003). Radiation-mediated proteolysis of CDT1 by CUL4-ROC1 and CSN complexes constitutes a new checkpoint. Nat. Cell Biol..

[113] Abbas T., Sivaprasad U., Terai K., Amador V., Pagano M., Dutta A. (2008). PCNA-dependent regulation of p21 ubiquitylation and degradation via the CRL4Cdt2 ubiquitin ligase complex. Genes Dev..

[114] Hu J., McCall C.M., Ohta T., Xiong Y. (2004). Targeted ubiquitination of CDT1 by the DDB1-CUL4A-ROC1 ligase in response to DNA damage. Nat. Cell Biol..

[115] Hannah J., Zhou P. (2009). Regulation of DNA damage response pathways by the cullin-RING ubiquitin ligases. DNA Repair.

[116] Lin J.J., Milhollen M.A., Smith P.G., Narayanan U., Dutta A. (2010). NEDD8-targeting drug MLN4924 elicits DNA rereplication by stabilizing Cdt1 in S phase, triggering checkpoint activation, apoptosis, and senescence in cancer cells. Cancer Res..

[117] Seiler J.A., Conti C., Syed A., Aladjem M.I., Pommier Y. (2007). The intra-S-phase checkpoint affects both DNA replication initiation and elongation: Single-cell and -DNA fiber analyses. Mol. Cell Biol..

[118] Zhao H., Zhang S., Xu D., Lee M.Y., Zhang Z., Lee E.Y., Darzynkiewicz Z. (2014). Expression of the p12 subunit of human DNA polymerase δ (Pol δ), CDK inhibitor p21(WAF1), Cdt1, cyclin A, PCNA and Ki-67 in relation to DNA replication in individual cells. Cell Cycle.

[119] Darzynkiewicz Z., Zhao H., Zhang S., Lee M.Y., Lee E.Y., Zhang Z. (2015). Initiation and termination of DNA replication during S phase in relation to cyclins D1, E and A, p21WAF1, Cdt1 and the p12 subunit of DNA polymerase δ revealed in individual cells by cytometry. Oncotarget.

[120] Balakrishnan L., Bambara R.A. (2013). Okazaki fragment metabolism. Cold Spring Harb. Perspect. Biol..

[121] Garg P., Stith C.M., Sabouri N., Johansson E., Burgers P.M. (2004). Idling by DNA polymerase δ maintains a ligatable nick during lagging-strand DNA replication. Genes Dev..

[122] Balakrishnan L., Bambara R.A. (2013). FLAP Endonuclease 1. Ann. Rev. Biochem..

[123] Jin Y.H., Ayyagari R., Resnick M.A., Gordenin D.A., Burgers P.M. (2003). Okazaki fragment maturation in yeast. II. Cooperation between the polymerase and 3′-5′-exonuclease activities of Pol δ in the creation of a ligatable nick. J. Biol. Chem..

[124] Burgers P.M. (2011). It’s all about flaps: DNA2 and checkpoint activation. Cell Cycle.

[125] Howes T.R., Tomkinson A.E. (2012). DNA ligase I, the replicative DNA ligase. Subcell. Biochem..

[126] Kelly R.B., Cozzarelli N.R., Deutscher M.P., Lehman I.R., Kornberg A. (1970). Enzymatic synthesis of deoxyribonucleic acid. XXXII. Replication of duplex deoxyribonucleic acid by polymerase at a single strand break. J. Biol. Chem..

[127] Lin S.H., Wang X., Zhang S., Zhang Z., Lee E.Y., Lee M.Y. (2013). Dynamics of Enzymatic Interactions during Short Flap Human Okazaki Fragment Processing by Two Forms of Human DNA Polymerase δ. DNA Repair.

[128] Beattie T.R., Bell S.D. (2012). Coordination of multiple enzyme activities by a single PCNA in archaeal Okazaki fragment maturation. EMBO J..

[129] Patel S.S., Wong I., Johnson K.A. (1991). Pre-steady-state kinetic analysis of processive DNA replication including complete characterization of an exonuclease-deficient mutant. Biochemistry.

[130] Langston L.D., O’Donnell M. (2008). DNA polymerase δ is highly processive with proliferating cell nuclear antigen and undergoes collision release upon completing DNA. J. Biol. Chem..

[131] Burgers P.M., Kunkel T.A. (2017). Eukaryotic DNA Replication Fork. Annu. Rev. Biochem..

[132] Huang Q.M., Akashi T., Masuda Y., Kamiya K., Takahashi T., Suzuki M. (2010). Roles of POLD4, smallest subunit of DNA polymerase δ, in nuclear structures and genomic stability of human cells. Biochem. Biophys. Res. Commun..

[133] Huang Q., Suzuki M., Zeng Y., Zhang H., Yang D., Lin H. (2014). Downregulation of POLD4 in Calu6 cells results in G1-S blockage through suppression of the Akt-Skp2-p27 pathway. Bioorg. Med. Chem. Lett..

[134] Liu L., Rodriguez-Belmonte E.M., Mazloum N., Xie B., Lee M.Y. (2003). Identification of a novel protein, PDIP38, that interacts with the p50 subunit of DNA polymerase δ and proliferating cell nuclear antigen. J. Biol. Chem..

[135] He H., Tan C.K., Downey K.M., So A.G. (2001). A tumor necrosis factor α- and interleukin 6-inducible protein that interacts with the small subunit of DNA polymerase δ and proliferating cell nuclear antigen. Proc. Natl. Acad. Sci. USA.

[136] Richardson C.J., Broenstrup M., Fingar D.C., Julich K., Ballif B.A., Gygi S., Blenis J. (2004). SKAR is a specific target of S6 kinase 1 in cell growth control. Curr. Biol..

[137] Magnuson B., Ekim B., Fingar D.C. (2012). Regulation and function of ribosomal protein S6 kinase (S6K) within mTOR signalling networks. Biochem. J..

[138] Richardson C.J., Schalm S.S., Blenis J. (2004). PI3-kinase and TOR: PIKTORing cell growth. Semin. Cell Dev. Biol..

[139] Banko M.I., Krzyzanowski M.K., Turcza P., Maniecka Z., Kulis M., Kozlowski P. (2013). Identification of amino acid residues of ERH required for its recruitment to nuclear speckles and replication foci in HeLa cells. PLoS ONE.

[140] Ma X.M., Yoon S.O., Richardson C.J., Julich K., Blenis J. (2008). SKAR links pre-mRNA splicing to mTOR/S6K1-mediated enhanced translation efficiency of spliced mRNAs. Cell.

[141] Ma X.M., Blenis J. (2009). Molecular mechanisms of mTOR-mediated translational control. Nat. Rev. Mol. Cell Biol..

[142] Fingar D.C., Salama S., Tsou C., Harlow E., Blenis J. (2002). Mammalian cell size is controlled by mTOR and its downstream targets S6K1 and 4EBP1/eIF4E. Genes Dev..

[143] Smyk A., Szuminska M., Uniewicz K.A., Graves L.M., Kozlowski P. (2006). Human enhancer of rudimentary is a molecular partner of PDIP46/SKAR, a protein interacting with DNA polymerase δ and S6K1 and regulating cell growth. FEBS J..

[144] Weng M.T., Luo J. (2013). The enigmatic ERH protein: Its role in cell cycle, RNA splicing and cancer. Protein Cell.

[145] Weng M.T., Tung T.H., Lee J.H., Wei S.C., Lin H.L., Huang Y.J., Wong J.M., Luo J., Sheu J.C. (2015). Enhancer of rudimentary homolog regulates DNA damage response in hepatocellular carcinoma. Sci. Rep..

[146] Kavanaugh G., Zhao R., Guo Y., Mohni K.N., Glick G., Lacy M.E., Hutson M.S., Ascano M., Cortez D. (2015). Enhancer of Rudimentary Homolog affects the replication stress response through regulation of RNA processing. Mol. Cell Biol..

[147] Gilljam K.M., Feyzi E., Aas P.A., Sousa M.M., Muller R., Vagbo C.B., Catterall T.C., Liabakk N.B., Slupphaug G., Drablos F. (2009). Identification of a novel, widespread, and functionally important PCNA-binding motif. J. Cell Biol..

[148] Gilljam K.M., Muller R., Liabakk N.B., Otterlei M. (2012). Nucleotide excision repair is associated with the replisome and its efficiency depends on a direct interaction between XPA and PCNA. PLoS ONE.

[149] Bacquin A., Pouvelle C., Siaud N., Perderiset M., Salome-Desnoulez S., Tellier-Lebegue C., Lopez B., Charbonnier J.B., Kannouche P.L. (2013). The helicase FBH1 is tightly regulated by PCNA via CRL4(Cdt2)-mediated proteolysis in human cells. Nucleic Acids Res..

[150] Fu D., Samson L.D., Hubscher U., van Loon B. (2015). The interaction between ALKBH2 DNA repair enzyme and PCNA is direct, mediated by the hydrophobic pocket of PCNA and perturbed in naturally-occurring ALKBH2 variants. DNA Repair.

[151] O’Donnell M., Li H. (2016). The Eukaryotic Replisome Goes Under the Microscope. Curr. Biol..

[152] Kang Y.H., Farina A., Bermudez V.P., Tappin I., Du F., Galal W.C., Hurwitz J. (2013). Interaction between human Ctf4 and the Cdc45/Mcm2-7/GINS (CMG) replicative helicase. Proc. Natl. Acad. Sci. USA.

[153] Villa F., Simon A.C., Ortiz Bazan M.A., Kilkenny M.L., Wirthensohn D., Wightman M., Matak-Vinkovic D., Pellegrini L., Labib K. (2016). Ctf4 Is a Hub in the Eukaryotic Replisome that Links Multiple CIP-Box Proteins to the CMG Helicase. Mol. Cell.

[154] Xie B., Li H., Wang Q., Xie S., Rahmeh A., Dai W., Lee M.Y. (2005). Further characterization of human DNA polymerase δ interacting protein 38. J. Biol. Chem..

[155] Pursell Z.F., Isoz I., Lundstrom E.B., Johansson E., Kunkel T.A. (2007). Yeast DNA polymerase epsilon participates in leading-strand DNA replication. Science.

[156] Nick McElhinny S.A., Gordenin D.A., Stith C.M., Burgers P.M., Kunkel T.A. (2008). Division of labor at the eukaryotic replication fork. Mol. Cell.

[157] Larrea A.A., Lujan S.A., Nick McElhinny S.A., Mieczkowski P.A., Resnick M.A., Gordenin D.A., Kunkel T.A. (2010). Genome-wide model for the normal eukaryotic DNA replication fork. Proc. Natl. Acad. Sci. USA.

[158] Johnson R.E., Klassen R., Prakash L., Prakash S. (2015). A Major Role of DNA Polymerase δ in Replication of Both the Leading and Lagging DNA Strands. Mol. Cell.

[159] Eckert K.A., Hile S.E. (2009). Every microsatellite is different: Intrinsic DNA features dictate mutagenesis of common microsatellites present in the human genome. Mol. Carcinog..

[160] Le H.P., Masuda Y., Tsurimoto T., Maki S., Katayama T., Furukohri A., Maki H. (2015). Short CCG repeat in huntingtin gene is an obstacle for replicative DNA polymerases, potentially hampering progression of replication fork. Genes Cells.

[161] Hile S.E., Wang X., Lee M.Y., Eckert K.A. (2012). Beyond translesion synthesis: Polymerase к fidelity as a potential determinant of microsatellite stability. Nucleic Acids Res..

[162] Baptiste B.A., Eckert K.A. (2012). DNA polymerase к microsatellite synthesis: Two distinct mechanisms of slippage-mediated errors. Environ. Mol. Mutagen..

[163] Rey L., Sidorova J.M., Puget N., Boudsocq F., Biard D.S., Monnat R.J.J., Cazaux C., Hoffmann J.S. (2009). Human DNA polymerase eta is required for common fragile site stability during unperturbed DNA replication. Mol. Cell Biol..

[164] Bergoglio V., Boyer A.S., Walsh E., Naim V., Legube G., Lee M.Y., Rey L., Rosselli F., Cazaux C., Eckert K.A. (2013). DNA synthesis by Pol eta promotes fragile site stability by preventing under-replicated DNA in mitosis. J. Cell Biol..

[165] Garcia-Exposito L., Bournique E., Bergoglio V., Bose A., Barroso-Gonzalez J., Zhang S., Roncaioli J.L., Lee M., Wallace C.T., Watkins S.C. (2016). Proteomic Profiling Reveals a Specific Role for Translesion DNA Polymerase eta in the Alternative Lengthening of Telomeres. Cell Rep..

[166] Bermudez V.P., Farina A., Raghavan V., Tappin I., Hurwitz J. (2011). Studies on Human DNA Polymerase epsilon and GINS Complex and Their Role in DNA Replication. J. Biol. Chem..

[167] Ganai R.A., Zhang X.P., Heyer W.D., Johansson E. (2016). Strand displacement synthesis by yeast DNA polymerase epsilon. Nucleic Acids Res..

[168] Yeeles J.T., Janska A., Early A., Diffley J.F. (2017). How the Eukaryotic Replisome Achieves Rapid and Efficient DNA Replication. Mol. Cell.

[169] Zhou J.C., Janska A., Goswami P., Renault L., Abid Ali F., Kotecha A., Diffley J.F.X., Costa A. (2017). CMG-Pol epsilon dynamics suggests a mechanism for the establishment of leading-strand synthesis in the eukaryotic replisome. Proc. Natl. Acad. Sci. USA.

[170] Kurat C.F., Yeeles J.T., Patel H., Early A., Diffley J.F. (2017). Chromatin Controls DNA Replication Origin Selection, Lagging-Strand Synthesis, and Replication Fork Rates. Mol. Cell.

[171] Yeeles J.T., Deegan T.D., Janska A., Early A., Diffley J.F. (2015). Regulated eukaryotic DNA replication origin firing with purified proteins. Nature.

[172] Schauer G.D., O’Donnell M.E. (2017). Quality control mechanisms exclude incorrect polymerases from the eukaryotic replication fork. Proc. Natl. Acad. Sci. USA.

[173] Yurieva O., O’Donnell M. (2016). Reconstitution of a eukaryotic replisome reveals the mechanism of asymmetric distribution of DNA polymerases. Nucleus.

[174] Hedglin M., Pandey B., Benkovic S.J. (2016). Stability of the human polymerase δ holoenzyme and its implications in lagging strand DNA synthesis. Proc. Natl. Acad. Sci. USA.

[175] Beattie T.R., Kapadia N., Nicolas E., Uphoff S., Wollman A.J., Leake M.C., Reyes-Lamothe R. (2017). Frequent exchange of the DNA polymerase during bacterial chromosome replication. Elife.

[176] Li J.J., Kelly T.J. (1984). Simian virus 40 DNA replication in vitro. Proc. Natl. Acad. Sci. USA.

[177] Waga S., Stillman B. (1994). Anatomy of a DNA replication fork revealed by reconstitution of SV40 DNA replication in vitro. Nature.

[178] Rytkonen A.K., Vaara M., Nethanel T., Kaufmann G., Sormunen R., Laara E., Nasheuer H.P., Rahmeh A., Lee M.Y., Syvaoja J.E. (2006). Distinctive activities of DNA polymerases during human DNA replication. FEBS J..

[179] Friedberg E.C., Lehmann A.R., Fuchs R.P. (2005). Trading places: How do DNA polymerases switch during translesion DNA synthesis?. Mol. Cell.

[180] Lehmann A.R. (2006). Translesion synthesis in mammalian cells. Exp. Cell Res..

[181] Guilliam T.A., Doherty A.J. (2017). PrimPol-Prime Time to Reprime. Genes.

[182] Khan F.H., Pandian V., Ramraj S., Natarajan M., Aravindan S., Herman T.S., Aravindan N. (2015). Acquired genetic alterations in tumor cells dictate the development of high-risk neuroblastoma and clinical outcomes. BMC Cancer.

[183] Human Protein Atlas. http://www.proteinatlas.org/ENSG00000100227-POLDIP3/cancer.

[184] COSMIC (Catalogue of Somatic Mutations in Cancer). http://cancer.sanger.ac.uk/cosmic.

[185] Gakh O., Cavadini P., Isaya G. (2002). Mitochondrial processing peptidases. Biochim. Biophys. Acta.

[186] Cheng X., Kanki T., Fukuoh A., Ohgaki K., Takeya R., Aoki Y., Hamasaki N., Kang D. (2005). PDIP38 associates with proteins constituting the mitochondrial DNA nucleoid. J. Biochem..

[187] Arakaki N., Nishihama T., Kohda A., Owaki H., Kuramoto Y., Abe R., Kita T., Suenaga M., Himeda T., Kuwajima M. (2006). Regulation of mitochondrial morphology and cell survival by Mitogenin I and mitochondrial single-stranded DNA binding protein. Biochim. Biophys. Acta.

[188] Klaile E., Muller M.M., Kannicht C., Otto W., Singer B.B., Reutter W., Obrink B., Lucka L. (2007). The cell adhesion receptor carcinoembryonic antigen-related cell adhesion molecule 1 regulates nucleocytoplasmic trafficking of DNA polymerase δ-interacting protein 38. J. Biol. Chem..

[189] Klaile E., Kukalev A., Obrink B., Muller M.M. (2008). PDIP38 is a novel mitotic spindle-associated protein that affects spindle organization and chromosome segregation. Cell Cycle.

[190] Tissier A., Janel-Bintz R., Coulon S., Klaile E., Kannouche P., Fuchs R.P., Cordonnier A.M. (2010). Crosstalk between replicative and translesional DNA polymerases: PDIP38 interacts directly with Poleta. DNA Repair.

[191] Maga G., Crespan E., Markkanen E., Imhof R., Furrer A., Villani G., Hubscher U., van Loon B. (2013). DNA polymerase δ-interacting protein 2 is a processivity factor for DNA polymerase lambda during 8-oxo-7,8-dihydroguanine bypass. Proc. Natl. Acad. Sci. USA.

[192] Bebenek K., Pedersen L.C., Kunkel T.A. (2014). Structure-Function Studies of DNA Polymerase lambda. Biochemistry.

[193] Mentegari E., Kissova M., Bavagnoli L., Maga G., Crespan E. (2016). DNA Polymerases lambda and beta: The Double-Edged Swords of DNA Repair. Genes.

[194] Waters L.S., Minesinger B.K., Wiltrout M.E., D’Souza S., Woodruff R.V., Walker G.C. (2009). Eukaryotic translesion polymerases and their roles and regulation in DNA damage tolerance. Microbiol. Mol. Biol. Rev..

[195] Braithwaite E.K., Kedar P.S., Stumpo D.J., Bertocci B., Freedman J.H., Samson L.D., Wilson S.H. (2010). DNA polymerases beta and lambda mediate overlapping and independent roles in base excision repair in mouse embryonic fibroblasts. PLoS ONE.

[196] Braithwaite E.K., Kedar P.S., Lan L., Polosina Y.Y., Asagoshi K., Poltoratsky V.P., Horton J.K., Miller H., Teebor G.W., Yasui A. (2005). DNA polymerase lambda protects mouse fibroblasts against oxidative DNA damage and is recruited to sites of DNA damage/repair. J. Biol. Chem..

[197] Hubscher U., Maga G. (2011). DNA replication and repair bypass machines. Curr. Opin. Chem. Biol..

[198] Guilliam T.A., Bailey L.J., Brissett N.C., Doherty A.J. (2016). PolDIP2 interacts with human PrimPol and enhances its DNA polymerase activities. Nucleic Acids Res..

[199] Wong A., Zhang S., Mordue D., Wu J.M., Zhang Z., Darzynkiewicz Z., Lee E.Y., Lee M.Y. (2013). PDIP38 is translocated to the spliceosomes/nuclear speckles in response to UV-induced DNA damage and is required for UV-induced alternative splicing of MDM2. Cell Cycle.

[200] Spector D.L., Lamond A.I. (2011). Nuclear speckles. Cold Spring Harb. Perspect. Biol..

[201] Campalans A., Amouroux R., Bravard A., Epe B., Radicella J.P. (2007). UVA irradiation induces relocalisation of the DNA repair protein hOGG1 to nuclear speckles. J. Cell Sci..

[202] Dutertre M., Sanchez G., Barbier J., Corcos L., Auboeuf D. (2011). The emerging role of pre-messenger RNA splicing in stress responses: Sending alternative messages and silent messengers. RNA Biol..

[203] Giono L.E., Nieto Moreno N., Cambindo Botto A.E., Dujardin G., Munoz M.J., Kornblihtt A.R. (2016). The RNA Response to DNA Damage. J. Mol. Biol..

[204] Bartel F., Taubert H., Harris L.C. (2002). Alternative and aberrant splicing of MDM2 mRNA in human cancer. Cancer Cell.

[205] Chandler D.S., Singh R.K., Caldwell L.C., Bitler J.L., Lozano G. (2006). Genotoxic stress induces coordinately regulated alternative splicing of the p53 modulators MDM2 and MDM4. Cancer Res..

[206] Jeyaraj S., O’Brien D.M., Chandler D.S. (2009). MDM2 and MDM4 splicing: An integral part of the cancer spliceome. Front. Biosci..

[207] Okoro D.R., Rosso M., Bargonetti J. (2012). Splicing up mdm2 for cancer proteome diversity. Genes Cancer.

[208] Jones S.N., Hancock A.R., Vogel H., Donehower L.A., Bradley A. (1998). Overexpression of Mdm2 in mice reveals a p53-independent role for Mdm2 in tumorigenesis. Proc. Natl. Acad. Sci. USA.

[209] Matsumoto R., Tada M., Nozaki M., Zhang C.L., Sawamura Y., Abe H. (1998). Short alternative splice transcripts of the mdm2 oncogene correlate to malignancy in human astrocytic neoplasms. Cancer Res..

[210] Naro C., Bielli P., Pagliarini V., Sette C. (2015). The interplay between DNA damage response and RNA processing: the unexpected role of splicing factors as gatekeepers of genome stability. Front. Genet..

[211] Montecucco A., Biamonti G. (2013). Pre-mRNA processing factors meet the DNA damage response. Front. Genet..

[212] Lambeth J.D., Neish A.S. (2014). Nox enzymes and new thinking on reactive oxygen: A double-edged sword revisited. Annu. Rev. Pathol..

[213] Bedard K., Krause K.H. (2007). The NOX family of ROS-generating NADPH oxidases: Physiology and pathophysiology. Physiol. Rev..

[214] Lambeth J.D., Kawahara T., Diebold B. (2007). Regulation of Nox and Duox enzymatic activity and expression. Free Radic. Biol. Med..

[215] Brown D.I., Griendling K.K. (2015). Regulation of signal transduction by reactive oxygen species in the cardiovascular system. Circ. Res..

[216] Valko M., Leibfritz D., Moncol J., Cronin M.T., Mazur M., Telser J. (2007). Free radicals and antioxidants in normal physiological functions and human disease. Int J. Biochem. Cell Biol..

[217] Lambeth J.D. (2007). Nox enzymes, ROS, and chronic disease: An example of antagonistic pleiotropy. Free Radic. Biol. Med..

[218] Nisimoto Y., Diebold B.A., Cosentino-Gomes D., Lambeth J.D. (2014). Nox4: A hydrogen peroxide-generating oxygen sensor. Biochemistry.

[219] Lassegue B., Griendling K.K. (2010). NADPH Oxidases: Functions and Pathologies in the Vasculature. Arterioscler. Thromb. Vasc. Biol..

[220] Lyle A.N., Deshpande N.N., Taniyama Y., Seidel-Rogol B., Pounkova L., Du P., Papaharalambus C., Lassegue B., Griendling K.K. (2009). Poldip2, a novel regulator of Nox4 and cytoskeletal integrity in vascular smooth muscle cells. Circ. Res..

[221] Datla S.R., McGrail D.J., Vukelic S., Huff L.P., Lyle A.N., Pounkova L., Lee M., Seidel-Rogol B., Khalil M.K., Hilenski L.L. (2014). Poldip2 controls vascular smooth muscle cell migration by regulating focal adhesion turnover and force polarization. Am. J. Physiol. Heart Circ. Physiol..

[222] Sutliff R.L., Hilenski L.L., Amanso A.M., Parastatidis I., Dikalova A.E., Hansen L., Datla S.R., Long J.S., El-Ali A.M., Joseph G. (2013). Polymerase Delta Interacting Protein 2 Sustains Vascular Structure and Function. Arterioscler. Thromb. Vasc. Biol..

[223] Brown D.I., Lassegue B., Lee M., Zafari R., Long J.S., Saavedra H.I., Griendling K.K. (2014). Poldip2 knockout results in perinatal lethality, reduced cellular growth and increased autophagy of mouse embryonic fibroblasts. PLoS ONE.

